# Plant-based diets and incident cardiovascular disease and all-cause mortality in African Americans: A cohort study

**DOI:** 10.1371/journal.pmed.1003863

**Published:** 2022-01-05

**Authors:** Leah J. Weston, Hyunju Kim, Sameera A. Talegawkar, Katherine L. Tucker, Adolfo Correa, Casey M. Rebholz

**Affiliations:** 1 Department of Medicine, Johns Hopkins University School of Medicine, Baltimore, Maryland, United States of America; 2 Department of Epidemiology, Johns Hopkins Bloomberg School of Public Health, Baltimore, Maryland, United States of America; 3 Department of Exercise and Nutrition Sciences, Milken School of Public Health, George Washington University, Washington, District of Columbia, United States of America; 4 Department of Biomedical and Nutritional Sciences, University of Massachusetts Lowell, Lowell, Massachusetts, United States of America; 5 Department of Medicine, University of Mississippi Medical Center, Jackson, Mississippi, United States of America; 6 Jackson Heart Study, Jackson, Mississippi, United States of America; University of Cambridge, UNITED KINGDOM

## Abstract

**Background:**

Prior studies have documented lower cardiovascular disease (CVD) risk among people with a higher adherence to a plant-based dietary pattern. Non-Hispanic black Americans are an understudied group with high burden of CVD, yet studies of plant-based diets have been limited in this population.

**Methods and findings:**

We conducted an analysis of prospectively collected data from a community-based cohort of African American adults (*n* = 3,635) in the Jackson Heart Study (JHS) aged 21–95 years, living in the Jackson, Mississippi, metropolitan area, US, who were followed from 2000 to 2018. Using self-reported dietary data, we assigned scores to participants’ adherence to 3 plant-based dietary patterns: an overall plant-based diet index (PDI), a healthy PDI (hPDI), and an unhealthy PDI (uPDI). Cox proportional hazards models were used to estimate associations between plant-based diet scores and CVD incidence and all-cause mortality. Over a median follow-up of 13 and 15 years, there were 293 incident CVD cases and 597 deaths, respectively. After adjusting for sociodemographic characteristics (age, sex, and education) and health behaviors (smoking, alcohol intake, margarine intake, physical activity, and total energy intake), no significant association was observed between plant-based diets and incident CVD for overall PDI (hazard ratio [HR] 1.06, 95% CI 0.78–1.42, *p-*trend = 0.72), hPDI (HR 1.07, 95% CI 0.80–1.42, *p-*trend = 0.67), and uPDI (HR 0.95, 95% CI 0.71–1.28, *p-*trend = 0.76). Corresponding HRs (95% CIs) for all-cause mortality risk with overall PDI, hPDI, and uPDI were 0.96 (0.78–1.18), 0.94 (0.76–1.16), and 1.06 (0.86–1.30), respectively. Corresponding HRs (95% CIs) for incident coronary heart disease with overall PDI, hPDI, and uPDI were 1.09 (0.74–1.61), 1.11 (0.76–1.61), and 0.79 (0.52–1.18), respectively. For incident total stroke, HRs (95% CIs) for overall PDI, hPDI, and uPDI were 1.00 (0.66–1.52), 0.91 (0.61–1.36), and 1.26 (0.84–1.89) (*p-*trend for all tests > 0.05). Limitations of the study include use of self-reported dietary intake, residual confounding, potential for reverse causation, and that the study did not capture those who exclusively consume plant-derived foods.

**Conclusions:**

In this study of black Americans, we observed that, unlike in prior studies, greater adherence to a plant-based diet was not associated with CVD or all-cause mortality.

## Abstract 2

In a cohort study, Leah J. Weston and colleagues investigate the associations between consumption of plant-based diets and incident cardiovascular disease and all-cause mortality in African Americans.

## Author summary

**Why was this study done?:**

- Plant-based diets have been linked, largely through studies of “vegetarian” diets, with health benefits, including lower risk of heart disease; however, studies of these associations among more general populations have produced mixed results.
- Investigating plant-based dietary patterns has allowed researchers to study how levels of adherence to plant-based dietary patterns and the healthfulness of plant-based diets correlate with cardiovascular disease (CVD) risk.
- This study was conducted to expand the generalizability of conclusions about plant-based diets and CVD risk in African American men and women in the US who were following a southern dietary pattern.

**What did the researchers do and find?:**

- We used data on 3,635 African American adults from the Jackson Heart Study with a mean follow-up of 13 years to assess the association of 3 plant-based dietary patterns (overall, healthy, and unhealthy) with CVD incidence and all-cause mortality.
- Overall diet quality was low for all participants, and participants with the most plant-rich diets still regularly included animal-based foods.
- Incidence of CVD and all-cause mortality was the same among participants whose diets were most similar to a plant-based dietary pattern and among those whose diets were least plant-based.
- Among individual food groups, legumes were associated with a lower risk for CVD, while vegetable oils were associated with higher risk for CVD, and whole grains and sugar-sweetened beverages were associated with higher all-cause mortality.

**What do these findings mean?:**

- Plant-based diet index scores have gained popularity among researchers for their ability to characterize overall dietary patterns; however, their use may be limited in populations with minimal gradations in the plant richness of participants’ diets.
- Health benefits linked to plant-based diets may depend on the overall healthfulness of an individual’s diet and might require higher levels of adherence to a plant-based diet than what is typically practiced among those with the most plant-rich diets in this cohort of African Americans residing in the southern region of the US.

## Introduction

Plant-based diets are gaining attention as more studies suggest both health and environmental sustainability benefits of dietary patterns characterized by lower meat consumption and higher consumption of fruit, vegetables, legumes, whole grains, nuts, and seeds [1]. Although observational studies have consistently found that vegetarians and vegans tend to have lower cardiometabolic risk factors and lower risk of heart disease, diabetes, kidney disease, and some cancers, there have been mixed findings among prospective studies investigating the association of plant-based diets with cardiovascular disease (CVD) and CVD risk factors [2–5]. These conflicting findings may be related to the attributes of the populations studied and variability in the healthiness of the vegetarian or vegan diets studied. Many cohorts have specifically recruited vegetarians, vegans, and health-conscious controls [5]. These groups tend to differ from the general population in several factors, including sociodemographics and health behaviors, which may limit the comparability and generalizability of these studies to the general US population [5,6].

To better address the possibility that these contrasting findings may be due to variability in the underlying healthfulness of participants’ diets, more recent studies have investigated plant-based diets in populations with wider generalizability [7–9]. In addition, rather than studying diets based on complete exclusion of food groups (i.e., vegetarian or vegan), there has been a trend toward characterizing diets based on relative adherence to a plant-based diet and to consider both unhealthy and healthy plant-based diet patterns. Diet indices reduce variability, contextualize the meaning of study findings, and allow for replication of the same scoring system in different study populations. However, not all of these large cohort studies are consistent in the magnitude or significance of their findings with respect to CVD incidence and mortality [7–9].

One limitation of existing research on plant-based diets is that this research may not adequately capture the dietary patterns of all Americans, particularly African Americans, who remain an understudied population with regard to plant-based dietary patterns. In a subgroup analysis of 592 black Americans (75% African Americans and 25% West Indians) in the Adventist Health Study published in 2015, vegetarians had lower odds of cardiometabolic risk factors compared with nonvegetarians, similar to in the overall Adventist Health Study cohort [10]. It has been reported that the prevalence of CVD is lower among black Americans in the Adventist Health Study compared to the overall US black American population. Also, the Adventist Health Study has limited generalizability, given differences in other influential health and lifestyle factors [10].

As one of the largest community-based cohorts of African American adults in the US, the Jackson Heart Study (JHS) provides a unique opportunity to investigate the association of plant-based diets with CVD morbidity and mortality [11]. The aim of this study is to evaluate whether 3 plant-based dietary patterns—an overall plant-based diet, a healthy plant-based diet, and an unhealthy plant-based diet—are associated with the risk of incident CVD or all-cause mortality in a southern African American population. Studying this cohort will allow us to increase the certainty and generalizability of conclusions on plant-based diets and CVD in African Americans, and expand our understanding of plant-based diets.

## Methods

### Study design

We conducted an analysis of prospectively collected data from the JHS, a longitudinal cohort study investigating CVD risk in African American individuals, aged 21–95 years, in Jackson, Mississippi [11]. Details of the study design, recruitment procedures, and measures have been published elsewhere [11–13]. The institutional review boards at Jackson State University, Tougaloo College, and the University of Mississippi Medical Center reviewed the protocol, and participants provided written informed consent. Participants underwent baseline assessments between 2000 and 2004 during which researchers conducted physical examinations and laboratory studies and collected data on medical history, medications, sociodemographic factors, and behavioral risk factors. This study is reported as per the Strengthening the Reporting of Observational Studies in Epidemiology (STROBE) guideline (S1 STROBE Checklist).

**Fig 1:**
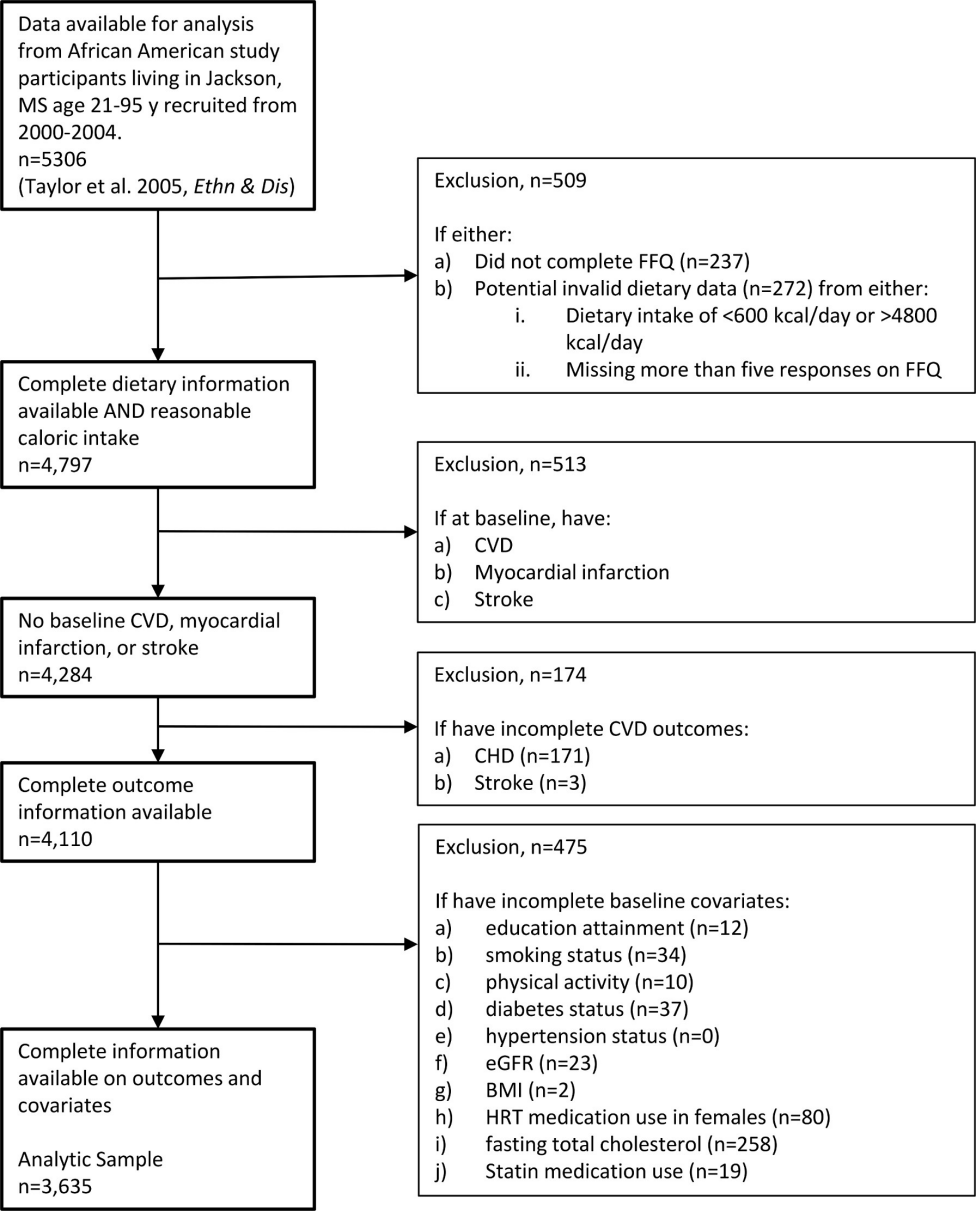
Participant selection flowchart. There were no missing data for age, sex, and total energy intake. Abbreviations: BMI, body mass index; CVD; cardiovascular disease; eGFR, estimated glomerular filtration rate; FFQ, food frequency questionnaire; HRT, hormone replacement therapy; MS, Mississippi.

The study enrolled 5,306 participants at baseline. We excluded 509 participants who did not complete the food frequency questionnaire (FFQ) (*n* = 237) or who had invalid or unavailable dietary data (*n* = 272) (defined as extremely low or high energy intake [<600 or >4,800 kcal/day] or missing more than 5 responses on the FFQ), leaving 4,797 participants with valid dietary assessment (Fig 1). Participants were further excluded if they had CVD, myocardial infarction, or stroke at baseline (*n* = 513) or if they had incomplete outcome information (missing coronary heart disease [CHD] or stroke, *n* = 174). We also excluded participants if they had missing data on covariates (education attainment, smoking status, physical activity, alcohol intake, margarine intake, fasting total cholesterol, body mass index [BMI], hypertension, diabetes, estimated glomerular filtration rate [eGFR], hormone replacement therapy [HRT] in women, and statin use; *n* = 475), leaving a final analytic sample of 3,635 participants.

### Dietary assessment

Dietary intake was assessed using an interviewer-administered, culturally appropriate, and validated FFQ developed for the study population, administered at baseline [14,15]. The JHS FFQ was based on the Delta NIRI FFQ, developed from 24-hour recalls in Mississippi, US [14,16]. Participants were asked to self-report the frequency and portion size of 158 food items consumed over the previous year. The reproducibility and validity of the FFQ used in the JHS was studied in a subset of 499 JHS participants, comparing the FFQ to 24-hour dietary recall data that were collected at the initial clinic visit and at 4 subsequent monthly administrations beginning 1 month after the initial clinic visit [15]. Average daily intakes of foods in servings per day were calculated using University of Minnesota Nutrition Data System for Research (NDSR) software (version 5.0–35, 2004; Nutrition Coordinating Center, University of Minnesota, Minneapolis).

### Plant-based diet scores

We used established plant-based diet indices (PDIs) for the present study, generating an overall PDI, a healthy PDI (hPDI), and an unhealthy PDI (uPDI) [7,9,17]. In previous studies, another plant-based diet score was developed in a Mediterranean population, i.e., a provegetarian diet index [18,19]. Given that the present study was conducted in participants from the US, we conducted our analysis using the overall PDI, hPDI, and uPDI. All food items were derived using the NDSR software based on participants’ responses on the FFQ. Then, the food categories from the NDSR were sorted into 1 of 18 food groups (S1 Table). These 18 food groups were further categorized into broader categories of animal food groups (animal fats, dairy, eggs, meat, and fish and seafood), healthy plant food groups (whole grains, fruits, vegetables, nuts, legumes, vegetable oils, and tea and coffee), and less healthy plant food groups (refined grains, potatoes, fruit juices, sugar-sweetened beverages [SSBs], sweets and desserts, and miscellaneous unhealthy plant-based foods). To account for existing dietary patterns of our study population, we modified the original indices by adding a “miscellaneous unhealthy plant-based foods” category and excluding the “mixed animal-based foods” category. Miscellaneous unhealthy plant-based foods included fried fruits, fried vegetables, and vegetable-based savory snacks. We did not include a “mixed animal-based foods” category in this index because such foods were already categorized into a primary food group by the NDSR software (pizza was categorized as cheese, beef-based tomato sauce as beef, etc.). Healthful and unhealthful foods were categorized based on their reported associations in the literature with chronic conditions, including type 2 diabetes, CVD, obesity, and hypertension [7,9,17,18]. Notably, the relative healthfulness of different food groups is not accounted for in the indices because all food groups are given equal weight in the diet index scores. The trans-fat content of margarine has changed in recent years [20]. Therefore, we did not include margarine in the index, but instead controlled for margarine intake in multivariable models, consistent with the approach from prior publications [7, 9,17,18]. We also did not include alcohol in our index, and instead controlled for it in our multivariable models, similar to previous studies [7, 9,17,18].

Indices were calculated by computing energy-adjusted consumption of each of the 18 food groups using the residual method [21,22] and dividing the energy-adjusted values into quintiles, assigned a score from 1 to 5. For the overall PDI, quintiles with the greatest relative consumption of healthy and less healthy plant foods were assigned a score of 5, and quintiles with the least relative consumption of healthy and less healthy plant foods were assigned a score of 1, with middle quintiles assigned a score of 2, 3, or 4. Participants with the highest relative consumption of animal foods were given reverse scores, such that the highest relative animal food consumption quintile received a score of 1 and the lowest relative animal food consumption quintile received a score of 5.

For hPDI, only healthy plant foods received positive scores: Participants in the highest quintile of healthy plant food consumption received a score of 5 (positive score), while participants in the highest quintile of unhealthy plant food consumption and the highest quintile of animal food consumption received a score of 1 (reverse score). For the uPDI, only the less healthy plant foods received positive scores, such that participants in the highest quintile of unhealthy plant food consumption received a score of 5 (positive score), while participants in the highest quintiles of healthy plant food consumption and animal food consumption received a score of 1 (reverse score). Indices had a theoretical range of 18 to 90, where 18 represents the least possible adherence to the particular index and 90 represents the greatest possible adherence to the diet index. All PDIs were divided into tertiles for analysis.

### Life’s Simple 7 total score and Life’s Simple 7 healthy diet score

In addition to PDIs, we calculated Life’s Simple 7 total score to examine the baseline characteristics of participants and Life’s Simple 7 healthy diet score to examine the nutritional characteristics of the plant-based diet scores. Life’s Simple 7 total score is a composite score describing cardiovascular health ranging from 0 to 14 that sums American Heart Association poor (0), intermediate (1), or ideal (2) health scores for smoking, diet, physical activity level, BMI, blood pressure, total cholesterol, and fasting plasma glucose [23]. Life’s Simple 7 healthy diet score is a measure of adherence to 5 healthy diet factors with the score ranging from 0 (least healthy) to 5 (most healthy) [24]. Healthy diet score components are as follows: fruits and vegetables, ≥4.5 cups/day; fish, ≥2 3.5-ounce servings/week; fiber-rich whole grains (≥1.1 g of fiber per 10 g of carbohydrate), ≥3 1-ounce servings per day; sodium, ≤1,500 mg/day; and SSBs, <36 fluid ounces/week (≤450 kcal/week). Dietary recommendations are scaled according to a 2,000-kcal/day diet.

### Outcome assessment

Surveillance for CVD events and deaths began on September 26, 2000, and continued until May 31, 2018. Details of the identification and classification of CVD events and deaths has been described elsewhere [25]. Briefly, CVD illnesses and deaths were identified through a combination of standardized annual telephone follow-up interviews and surveillance of hospitalizations and death certificates with adjudication by trained medical professionals. Every year, participants’ contact information was verified to help maintain contact in the following year. All-cause mortality was defined as deaths attributable to any cause. Incident CVD was defined as any new CHD event (including fatal CHD, myocardial infarction, or cardiac procedure) or stroke event that occurred during the follow-up window in an individual without prior history of CVD, myocardial infarction, or stroke. We did not include incident heart failure in our measure of CVD because monitoring for heart failure hospitalization did not begin until 2005. For all outcomes, participants were censored at loss to follow-up or end of study, and for CVD incidence analysis, participants were additionally censored at death.

### Covariate assessment

Participants’ sociodemographic information (age, sex, and education), health behaviors (cigarette smoking, physical activity, total energy intake, alcohol use, and margarine intake), medical history (hypertension status, diabetes status, HRT use, and statin medication use), BMI, and laboratory information (total cholesterol and eGFR) were collected at baseline [11]. Trained staff measured participants’ height to the nearest centimeter and weight to the nearest 0.1 kilogram, which were used to calculate BMI (kg/m^2^). Physical activity level was measured using the JHS Physical Activity Cohort Survey [26]. Alcohol use, margarine intake, and total energy intake were estimated using data reported in the FFQ. Hypertension was defined as having blood pressure ≥ 140/90 mm Hg or use of blood-pressure-lowering medication within 2 weeks prior to the clinic visit. Diabetes was defined as having fasting glucose ≥ 7.0 mmol/L, having hemoglobin A1c (HbA1c) ≥ 6.5%, or use of diabetic medication within 2 weeks prior to the clinic visit. eGFR was assessed using the 4-variable Chronic Kidney Disease Epidemiology Collaboration equation [27].

### Statistical analyses

Differences in baseline characteristics and nutritional characteristics, according to tertiles of PDI scores (overall PDI, hPDI, and uPDI) were evaluated by the chi-squared test for categorical variables and ANOVA for continuous variables. We examined macro- and micro-nutrient intake and Life’s Simple 7 healthy diet score (a measure of adherence to 5 healthy dietary goals including higher intake of fruits and vegetables, fish, and whole grains, and lower intake of sodium and SSBs) to describe nutritional characteristics of each of the PDIs [24].

For the primary analysis, we used Cox proportional hazards regression models to describe associations between PDIs and incident CVD and all-cause mortality. Length of follow-up (time since baseline) was used as the time metric. We adjusted the analysis for potential confounders in 3 progressively adjusted models. In model 1, we adjusted for age, sex, and total energy intake (kcal/day). Model 2 was adjusted for all variables in model 1 and further for educational attainment (less than high school, high school or General Educational Development [GED], greater than high school), smoking status (current, former, never), physical activity (continuous), alcohol intake (g/day), and margarine intake (servings/day). Model 3 was adjusted for all variables in model 2 and further for diabetes (yes/no), hypertension (yes/no), total cholesterol (continuous), eGFR (continuous), BMI (continuous), HRT medication use (yes/no), and statin medication use (yes/no). We calculated *p-*trend to assess the linear trend of hazard ratios (HRs) in Cox proportional hazards regression models, using the median value of each diet score tertile. HRs and 95% CIs were calculated by tertile. Finally, we conducted stratified analyses to determine whether associations differed by sex, BMI category (18.5 to <25 kg/m^2^, 25 to <30 kg/m^2^, ≥30 kg/m^2^), hypertension status, or diabetes status.

As secondary analyses, we modeled (1) components within the PDIs (healthy plant foods, unhealthy plant foods, and animal foods) and (2) 18 individual food groups within the indices together, instead of the scores, to test if a specific component or food group was associated with CVD incidence and all-cause mortality. All analyses were conducted using Stata statistical software, version 16.1 (StataCorp, College Station, TX), and significance was defined as a 2-sided *p-*value < 0.05.

As post hoc analyses, we (1) analyzed plant-based diets as continuous variables (HR per 1 standard deviation higher), (2) analyzed CVD separately as CHD (*n* = 173) and stroke (*n* = 148), (3) analyzed stroke separately as ischemic (*n* = 135) and hemorrhagic stroke (*n* = 12), (4) examined if there were departures from linearity by formally testing for linear association and modeling plant-based diet scores using restricted cubic splines with 4 knots at the 5th, 35th, 65th, and 95th percentiles, (5) simultaneously adjusted for hPDI and uPDI, (6) divided plant-based diet scores into quintiles instead of tertiles, (7) compared the baseline characteristics of the participants in our analytic sample (*n* = 3,635) and the total eligible study population including those with missing covariates (*n* = 4,110), and (8) used multiple imputation by chained equations to impute missing covariates (educational attainment, smoking status, alcohol intake, margarine intake, physical activity, BMI, total cholesterol, diabetes, eGFR, HRT medication use, and statin medication use) to assess the robustness of our findings [28]. For the analysis of stroke subtypes, we excluded 1 participant with missing stroke subtype information. For hemorrhagic stroke, we examined plant-based diets only as a continuous variable, due to the small number of hemorrhagic stroke cases (*n* = 12).

## Results

### Baseline characteristics

The overall PDI ranged from 30 to 76, while hPDI ranged from 34 to 82 and the uPDI ranged from 30 to 76. Participants with higher overall PDI and hPDI were more likely to be older, female, more educated, and more physically active, and to have lower eGFR and higher Life’s Simple 7 total score. Participants with higher overall PDI were more likely to have lower total energy intake, to have lower alcohol intake, to be nonsmokers, to have higher fasting total cholesterol, to have lower BMI, and to use statin medication (Table 1). Participants with higher hPDI were more likely to have higher intake of total energy, alcohol, and margarine (S2 Table). Conversely, participants with higher uPDI were more likely to be younger and to be male, and to have higher intake of total energy and alcohol, lower educational attainment, lower physical activity, and lower overall Life’s Simple 7 total score (S3 Table). Those with higher uPDI were also less likely to have hypertension or diabetes, had lower BMI, and lower HbA1c (*p* < 0.05 for all comparisons). Baseline characteristics were similar for the participants included in our analyses and the total eligible study population including those with missing data (S4 Table). Imputing missing covariates did not substantially change the results (S5 Table).

**Table 1:** Selected baseline demographic, socioeconomic, and health characteristics by tertiles of plant-based diet index in the Jackson Heart Study.

Characteristic	Overall plant-based diet index			*p-*Value
	Tertile 1 *n* = 1,237 (34.0%)	Tertile 2 *n* = 1,258 (34.6%)	Tertile 3 *n* = 1,140 (31.4%)	
Median index score (range)	48 (30–51)	55 (52–57)	61 (58–76)	
Age, years	51.9 (12.8)	54.2 (12.6)	55.5 (12.1)	<0.001
Female	52.5	67.8	72.5	<0.001
Total energy intake, kcal/day	2,525 (899)	2,142 (916)	2,103 (836)	<0.001
Educational attainment				0.021
Less than high school/GED	16.2	15.1	14.2	
High school or GED completion	21.1	20.4	16.8	
Attended college or trade school	62.7	64.5	68.9	
Smoking status				<0.001
Current smoker, percent	14.4	10.6	8.4	
Former smoker, percent	20.0	15.8	17.1	
Physical activity index*	2.1 (0.8)	2.1 (0.8)	2.2 (0.8)	0.01
Alcohol intake, g/day	6.4 (18.0)	3.1 (9.2)	1.9 (6.3)	<0.001
Margarine intake, servings/day	1.4 (1.7)	1.2 (1.8)	1.2 (1.7)	0.052
Fasting total cholesterol, mmol/L	5.1 (1.0)	5.1 (1.0)	5.3 (1.0)	0.001
Hypertension^†^	49.7	53.3	54.3	0.059
Diabetes^‡^	20.9	18.2	17.1	0.053
eGFR, ml/min/1.73 m^2§^	98.3 (20.5)	95.5 (20.5)	94.5 (19.7)	<0.001
BMI, kg/m^2^	32.2 (7.4)	31.9 (7.3)	31.1 (6.8)	<0.001
HRT medication use, percent of females	14.4	19.9	19.5	0.507
Statin medication use	9.1	11.1	13.2	0.006
Systolic blood pressure, mm Hg	127 (16)	127 (17)	127 (17)	0.613
HbA1c, mmol/mol	41 (13.1)	40 (12.0)	40 (12.0)	0.141
Life’s Simple 7 total score^||^	6.9 (2.1)	7.2 (2.0)	7.3 (2.1)	<0.001

Values are mean (standard deviation) for continuous variables and percent for categorical variables, unless otherwise noted. Statistical differences by tertiles of plant-based diet index were tested using analysis of variance for continuous variables and chi-squared tests for categorical variables, with *p* < 0.05 denoting statistical significance.

*Physical activity index is a measure from 0 (low) to 5 (high) of activity in daily living.

^†^Hypertension was defined as having blood pressure ≥ 140/90 mm Hg or use of blood-pressure-lowering medication within 2 weeks prior to the clinic visit.

^‡^Diabetes was defined as having fasting glucose ≥ 7.0 mmol/L, having HbA1c ≥ 6.5%, or use of diabetic medication within 2 weeks prior to the clinic visit.

^§^eGFR was assessed using the 4-variable Chronic Kidney Disease Epidemiology Collaboration equation.

^||^Life’s Simple 7 total score is a composite score describing cardiovascular health ranging from 0 to 14 that sums American Heart Association poor (0), intermediate (1), or ideal (2) health scores for smoking, diet, physical activity level, BMI, blood pressure, total cholesterol, and fasting plasma glucose.

BMI, body mass index; eGFR, estimated glomerular filtration rate; GED, General Educational Development; HRT, hormone replacement therapy; HbA1c, hemoglobin A1c.

### Nutritional characteristics

Nutritional characteristics of the diet differed significantly across tertiles of plant-based diet scores (Tables 2, S6, and S7). Participants in the highest tertiles of overall PDI, hPDI, and uPDI, respectively, met an average of 1.5, 1.6, and 0.8 of the 5 diet metrics in the Life’s Simple 7 healthy diet score (*p-*values for all tests < 0.001). Participants in the highest tertile of overall PDI had slightly lower total energy intake than those in the lowest tertile of overall PDI, whereas participants in the highest tertile of hPDI had slightly higher total energy intake than those in the lowest tertile of hPDI (*p-*values for all tests < 0.001). There was no linear trend in total energy intake across uPDI tertiles.

**Table 2:** Selected nutritional characteristics by tertiles of plant-based diet index in the Jackson Heart Study.

Characteristic	Overall plant-based diet index			*p-*Value
	Tertile 1	Tertile 2	Tertile 3	
Life’s Simple 7 healthy diet score*	1.1 (1.0)	1.3 (0.9)	1.5 (0.9)	<0.001
Total energy intake, kcal/day	2,525 (899)	2,142 (916)	2,103 (836)	<0.001
Total fat, g/day	106.9 (42.9)	86.2 (40.9)	81.4 (38.1)	<0.001
Protein, g/day	98.2 (39.4)	76.6 (35.1)	69.2 (30.7)	<0.001
Alcohol, g/day	6.4 (17.6)	3.2 (8.6)	2.1 (6.3)	<0.001
Saturated fatty acid, g/day	35.5 (14.9)	26.6 (13.0)	23.2 (11.5)	<0.001
Carbohydrates, g/day	290 (117)	269 (123)	283 (115)	<0.001
Dietary fiber, g/day	21.1 (9.6)	21.4 (10.3)	24.7 (11.1)	<0.001
Fruit, servings/day	2.5 (2.7)	2.8 (2.9)	3.7 (3.3)	<0.001
Vegetables, servings/day	3.9 (2.4)	3.8 (2.4)	4.4 (2.7)	<0.001
Whole grains, servings/day	0.8 (0.8)	1.0 (0.9)	1.3 (1.0)	<0.001
Nuts, g/day	7.3 (11.5)	7.6 (10.8)	9.0 (11.7)	<0.001
Fish, g/day	24.5 (36.4)	19.7 (26.9)	18.9 (23.0)	<0.001
Processed meat, g/day	28.0 (31.1)	18.3 (20.5)	13.7 (15.5)	<0.001
Beverages, g/day	359 (369)	314 (330)	301 (327)	<0.001
Sweetened beverages, servings/week	12.9 (17.7)	11.0 (15.0)	9.9 (12.2)	<0.001
Animal protein, g/day	70.0 (32.9)	47.6 (25.7)	36.8 (21.3)	<0.001
Vegetable protein, g/day	23.5 (9.5)	22.0 (9.7)	23.3 (9.5)	<0.001
Cholesterol, mg/day	459 (218)	297 (163)	221 (127)	<0.001
Monounsaturated fatty acids, g/day	38.1 (16.3)	29.6 (15.5)	27.0 (14.1)	<0.001
Polyunsaturated fatty acids, g/day	22.0 (9.9)	18.9 (10.0)	18.8 (9.7)	<0.001
Sodium, mg/day	4,001 (1,533)	3,283 (1,461)	3,077 (1,343)	<0.001
Potassium, mg/day	2,781 (1,122)	2,448 (1,084)	2,421 (1,049)	<0.001
Phosphorus, mg/day	1,449 (570)	1,131 (508)	1,026 (454)	<0.001
Calcium, mg/day	894 (413)	717 (347)	647 (291)	<0.001
Magnesium, mg/day	281 (100)	250 (99)	257 (99)	<0.001
Iron, mg/day	14.9 (6.2)	12.8 (5.9)	12.7 (5.5)	<0.001
Vitamin A, mg/day	7,181 (3,268)	6,745 (3,396)	7,002 (3,047)	0.003
Vitamin C, mg/day	113 (83)	110 (79)	118 (75)	0.063
Folate, mg/day	296 (123)	266 (115)	277 (113)	<0.001
Vitamin B12, μg/day	6.7 (4.2)	4.9 (3.4)	4.0 (2.6)	<0.001
Zinc, mg/day	12.7 (5.6)	10.0 (4.8)	9.1 (4.3)	<0.001

Values are mean (standard deviation). Statistical differences were tested using analysis of variance for continuous variables with *p* < 0.05 denoting statistical significance. Dietary data were self-reported.

*Life’s Simple 7 healthy diet score is a measure of adherence to 5 healthy dietary goals with score ranging from 0 (least healthy) to 5 (most healthy). Healthy diet score components are as follows: fruits and vegetables, ≥4.5 cups/day; fish, ≥2 3.5-ounce servings/week; fiber-rich whole grains (≥1.1 g of fiber per 10 g of carbohydrate), ≥3 1-ounce servings per day; sodium, ≤1,500 mg/day; and sugar-sweetened beverages, <36 fluid ounces/week (≤450 kcal/week). Dietary recommendations are scaled according to a 2,000-kcal/day diet.

Participants in the highest versus lowest tertile of overall PDI and hPDI reported higher consumption of fruits and vegetables, whereas those in the highest versus lowest uPDI tertile reported lower consumption of fruits and vegetables (Tables 2, S6, and S7). Those in the highest versus lowest tertile of overall PDI consumed less animal protein, processed meat, saturated fatty acids, and SSBs (Table 2). Those in the highest versus lowest tertile of hPDI consumed less processed meat, and similar amounts of animal protein and SSBs (S6 Table). Those in the highest versus lowest tertile of uPDI consumed less animal protein, more SSBs, and similar amounts of processed meat (S7 Table).

### Plant-based diets and CVD incidence and all-cause mortality

During a median follow-up of 13 years, there were 293 observed cases of incident CVD. During a median follow-up time of 15 years, there were 597 observed deaths. Incidence rates for CVD and all-cause mortality did not differ by PDI score (overall PDI, hPDI, or uPDI) (S8 Table). In multivariable regression models, there were no significant differences in risk of incident CVD or all-cause mortality by tertiles of plant-based diet (Table 3). HRs and 95% CIs for CVD incidence in the highest versus lowest tertile of overall PDI, hPDI, and uPDI were 1.06 (95% CI 0.78–1.42, *p-*trend = 0.72), 1.07 (95% CI 0.80–1.42, *p-*trend = 0.67), and 0.95 (95% CI 0.71–1.28, *p-*trend = 0.76), respectively, after adjusting for age, sex, energy intake, education, smoking, physical activity, and alcohol and margarine intake (model 2). A standard deviation increase in overall PDI (HR 1.05, 95% CI 0.93–1.20), hPDI (HR 1.07, 95% CI 0.95–1.20), and uPDI (HR 1.01, 95% CI 0.89–1.14) was not associated with incident CVD. HRs for all-cause mortality risk for the highest versus lowest tertile of overall PDI, hPDI, and uPDI were 0.96 (95% CI 0.78–1.18, *p-*trend = 0.67), 0.94 (95% CI 0.76–1.16, *p-*trend = 0.63), and 1.06 (95% CI 0.86–1.30, *p-*trend = 0.59), respectively. In a model adjusting for age, sex, and total energy intake (model 1), a standard deviation increase in hPDI was inversely associated with all-cause mortality (HR 0.89, 95% CI 0.82–0.97), whereas uPDI was positively associated with all-cause mortality (HR 1.09, 95% CI 1.00–1.19). However, these associations were attenuated in the most adjusted model (model 3) (hPDI: HR 0.93, 95% CI 0.85–1.02; uPDI: HR 1.09, 95% CI 1.00–1.19).

**Table 3:** Hazard ratios (95% confidence intervals) of incident cardiovascular disease and all-cause mortality among dietary patterns for progressively adjusted models.

Dietary index	Measure	Incident cardiovascular disease						All-cause mortality					
		Tertile 1 (ref)	Tertile 2	Tertile 3	*p-*Trend	Per SD higher score	*p-*Value	Tertile 1 (ref)	Tertile 2	Tertile 3	*p-*Trend	Per SD higher score	*p-*Value
Overall plant-based diet index	Median score	48	55	61				48	55	61			
	Cases/*N*	90/1,237	103/1,258	100/1,140				194/1,237	203/1,258	200/1,140			
	Person-years	14,507	14,787	13,470				17,998	18,412	16,659			
	HR (95% CI)												
	Model 1	1	1.02 (0.76–1.36)	1.06 (0.79–1.43)	0.69	1.05 (0.93, 1.19)	0.40	1	0.87 (0.71–1.06)	0.93 (0.76–1.14)	0.49	0.97 (0.89, 1.05)	0.44
	Model 2	1	0.98 (0.73–1.31)	1.06 (0.78–1.42)	0.72	1.05 (0.93, 1.20)	0.41	1	0.86 (0.70–1.05)	0.96 (0.78–1.18)	0.67	0.98 (0.89, 1.08)	0.72
	Model 3	1	1.02 (0.75–1.37)	1.09 (0.80–1.47)	0.60	1.07 (0.94, 1.22)	0.30	1	0.93 (0.76–1.15)	1.07 (0.87–1.33)	0.52	1.03 (0.95, 1.14)	0.45
Healthy plant-based diet index	Median score	48	54	60				48	54	60			
	Cases/*N*	101/1,295	91/1,155	101/1,185				201/1,295	226/1,155	170/1,185			
	Person-years	15,179	13,531	14,054				18,906	16,644	17,518			
	HR (95% CI)												
	Model 1	1	0.97 (0.73–1.29)	1.06 (0.80–1.41)	0.68	1.06 (0.94, 1.19)	0.36	1	1.24 (1.02–1.50)	0.87 (0.70–1.07)	0.21	0.89 (0.82, 0.97)	0.008
	Model 2	1	0.97 (0.73–1.30)	1.07 (0.80–1.42)	0.67	1.07 (0.95, 1.20)	0.27	1	1.27 (1.05–1.55)	0.94 (0.76–1.16)	0.63	0.93 (0.85, 1.01)	0.095
	Model 3	1	0.97 (0.72–1.29)	1.02 (0.76–1.36)	0.92	1.04 (0.92, 1.18)	0.52	1	1.29 (1.06–1.57)	0.94 (0.76–1.17)	0.65	0.93 (0.85, 1.02)	0.11
Unhealthy plant-based diet index	Median score	48	54	61				48	54	61			
	Cases/*N*	103/1,289	109/1,247	81/1,099				195/1,289	221/1,247	181/1,099			
	Person-years	15,183	14,536	13,045				18,869	18,103	16,097			
	HR (95% CI)												
	Model 1	1	1.08 (0.82–1.41)	0.97 (0.72–1.30)	0.85	1.02 (0.90, 1.15)	0.76	1	1.17 (0.96–1.42)	1.14 (0.93–1.40)	0.21	1.09 (1.00, 1.19)	0.045
	Model 2	1	1.06 (0.81–1.40)	0.95 (0.71–1.28)	0.76	1.01 (0.89, 1.14)	0.93	1	1.10 (0.90–1.34)	1.06 (0.86–1.30)	0.59	1.04 (0.96, 1.14)	0.31
	Model 3	1	1.01 (0.83–1.45)	1.03 (0.76–1.40)	0.82	1.05 (0.93, 1.19)	0.42	1	1.19 (0.98–1.46)	1.15 (0.93–1.42)	0.20	1.09 (1.00, 1.19)	0.047

Dietary data were self-reported. Incident cardiovascular disease is a composite of coronary heart disease and/or stroke events. Model 1 was adjusted for age, sex, and total energy intake. Model 2 was adjusted for all the covariates in model 1 and was further adjusted for educational attainment, smoking status, alcohol intake, margarine intake, and physical activity. Model 3 was adjusted for all the covariates in model 2 and was further adjusted for body mass index, total cholesterol, hypertension, diabetes, estimated glomerular filtration rate, hormone replacement therapy medication use, and statin medication use. Standard deviation (SD) for the overall plant-based diet index was 6.7, SD for the healthy plant-based diet index was 6.0, and SD for the unhealthy plant-based diet index was 6.7.

When CVD was analyzed separately, we found no association between any of the PDIs and incident CHD (*p-*trend for all tests > 0.05) (S9 Table), hPDI was inversely associated with ischemic stroke (HR 0.86, 95% CI 0.56–1.32), and uPDI was positively associated with ischemic stroke (HR 1.17, 95% CI 0.77–1.79), but none of these associations were statistically significant (*p-*trend for all tests > 0.05) (S10 Table). No significant association was observed for plant-based diet scores and hemorrhagic stroke (*p-*values for all tests > 0.05). We did not find departures from linearity when we tested for nonlinear associations for CVD and all-cause mortality (*p* for nonlinear association > 0.05 for all indices) or when we examined the shape of the association using restricted cubic splines (S1–S6 Figs). Simultaneously adjusting for hPDI and uPDI (range of HRs for hPDI and uPDI 0.96–1.09, *p-*trend for all tests > 0.05) or using quintiles instead of tertiles did not change the results for incident CVD or all-cause mortality (S11 Table).

Results for population subgroups, by sex, BMI, hypertension status, and diabetes status were similar to the main results, and there was no difference in association by subgroups (*p-*interaction > 0.05 for all tests) (Table 4).

**Table 4:** Adjusted hazard ratios (95% CIs) for incident cardiovascular disease and all-cause mortality for highest versus lowest tertile of plant-based diet scores according to sex and baseline diabetes status, hypertension status, and body mass index (BMI).

Dietary index	Subgroup	Incident cardiovascular disease		All-cause mortality	
		Hazard ratio (95% CI)	*p-*Interaction	Hazard ratio (95% CI)	*p-*Interaction
Overall plant-based diet index	Overall	1.09 (0.80–1.47)	0.60	1.07 (0.87–1.33)	0.52
	Females	1.02 (0.99–1.05)	0.13	1.02 (0.99–1.04)	0.20
	Males	0.99 (0.95–1.03)		0.99 (0.96–1.02)	
	BMI 18.5 to <25 kg/m^2^	0.96 (0.89–1.03)	0.29	1.04 (0.99–1.09)	0.30
	BMI 25 to <30 kg/m^2^	0.99 (0.95–1.03)		0.99 (0.96–1.03)	
	BMI ≥ 30 kg/m^2^	1.03 (1.00–1.06)		1.01 (0.98–1.03)	
	Diabetes	1.04 (1.00–1.09)	0.18	1.02 (0.99–1.05)	0.38
	No diabetes	0.99 (0.96–1.02)		1.00 (0.98–1.02)	
	Hypertension	1.01 (0.99–1.04)	0.77	1.00 (0.98–1.02)	0.52
	No hypertension	0.99 (0.93–1.04)		1.01 (0.98–1.05)	
Healthy plant-based diet index	Overall	1.02 (0.76–1.36)	0.92	0.94 (0.76–1.17)	0.65
	Females	1.01 (0.98–1.04)	0.47	1.00 (0.98–1.03)	0.08
	Males	0.99 (0.95–1.03)		0.98 (0.95–1.01)	
	BMI 18.5 to <25 kg/m^2^	0.99 (0.92–1.07)	0.18	1.04 (0.99–1.09)	0.14
	BMI 25 to <30 kg/m^2^	0.97 (0.93–1.02)		1.00 (0.97–1.03)	
	BMI ≥ 30 kg/m^2^	1.02 (0.99–1.06)		0.98 (0.96–1.01)	
	Diabetes	1.01 (0.96–1.05)	0.94	0.99 (0.96–1.02)	0.92
	No diabetes	1.00 (0.97–1.03)		1.00 (0.98–1.02)	
	Hypertension	1.01 (0.99–1.04)	0.09	0.99 (0.97–1.01)	0.50
	No hypertension	0.95 (0.89–1.00)		1.01 (0.97–1.05)	
Unhealthy plant-based diet index	Overall	0.97 (0.72–1.30)	0.85	1.15 (0.93–1.42)	0.20
	Females	1.00 (0.97–1.03)	0.80	1.01 (0.99–1.03)	0.99
	Males	1.00 (0.96–1.04)		1.01 (0.98–1.04)	
	BMI 18.5 to <25 kg/m^2^	0.98 (0.92–1.05)	0.87	0.99 (0.95–1.03)	0.36
	BMI 25 to <30 kg/m^2^	1.01 (0.97–1.05)		1.00 (0.97–1.03)	
	BMI ≥ 30 kg/m^2^	1.00 (0.96–1.03)		1.02 (1.00–1.04)	
	Diabetes	1.00 (0.96–1.04)	0.78	1.03 (1.00–1.06)	0.27
	No diabetes	1.00 (0.98–1.03)		1.00 (0.99–1.02)	
	Hypertension	0.99 (0.97–1.02)	0.09	1.01 (1.00–1.03)	0.46
	No hypertension	1.04 (0.99–1.10)		1.00 (0.96–1.03)	

Dietary data were self-reported. Models adjusted for age, sex, total energy intake, educational attainment, smoking status, alcohol intake, margarine intake, physical activity, body mass index (BMI), total cholesterol, hypertension, diabetes, estimated glomerular filtration rate, hormone replacement therapy medication use, and statin medication use.

### Analyses on score components and individual food groups

We found no significant association between score components (healthy plant-based foods, unhealthy plant-based foods, and animal-based foods) and incident CVD or all-cause mortality when controlling for all covariates and other score components (S12 Table). In the analysis of individual food groups, we observed significant associations, per 1-serving increase, of whole grain consumption with all-cause mortality (HR 1.13, 95% CI 1.02–1.25), SSB consumption with all-cause mortality (HR 1.07, 95% CI 1.00–1.14), legume consumption with lower CVD risk (HR 0.59, 95% CI 0.35–0.99), and healthy oil consumption with higher CVD risk (HR 1.10, 95% CI 1.01–1.20) after adjusting for covariates and all other individual food groups (Table 5).

**Table 5:** Adjusted hazard ratios (95% CIs) for incident cardiovascular disease and all-cause mortality per 1-serving increase in individual food groups.

Food group	Incident cardiovascular disease		All-cause mortality	
	Hazard ratio (95% CI)	*p-*Trend	Hazard ratio (95% CI)	*p-*Trend
Healthy plant foods				
Whole grains	1.03 (0.89–1.19)	0.71	1.13 (1.02–1.25)	0.019
Vegetables	1.17 (0.96–1.41)	0.12	1.00 (0.86–1.16)	0.99
Fruits	0.87 (0.72–1.06)	0.16	1.03 (0.97–1.09)	0.34
Nuts	0.88 (0.71–1.08)	0.23	0.92 (0.79–1.06)	0.24
Legumes	0.59 (0.35–0.99)	0.047	0.71 (0.50–1.00)	0.053
Healthy vegetable oils	1.10 (1.01–1.20)	0.019	1.03 (0.96–1.10)	0.40
Unsweetened coffee and tea	1.02 (0.95–1.08)	0.62	1.02 (0.97–1.07)	0.53
Unhealthy plant foods				
Refined grains	1.05 (0.94–1.16)	0.39	1.05 (0.97–1.13)	0.21
Potatoes	1.19 (0.85–1.68)	0.30	1.11 (0.86–1.44)	0.41
Fruit juice	1.01 (0.92–1.12)	0.77	1.05 (0.98–1.12)	0.15
Sugar-sweetened beverages	1.06 (0.97–1.16)	0.21	1.07 (1.00–1.14)	0.045
Sweets and desserts	0.98 (0.94–1.02)	0.32	0.99 (0.96–1.02)	0.43
Miscellaneous unhealthy plant-based foods	1.07 (0.77–1.48)	0.69	1.21 (0.97–1.51)	0.10
Animal-based foods				
Animal fat	0.95 (0.85–1.07)	0.39	1.05 (0.97–1.12)	0.22
Dairy	1.03 (0.88–1.21)	0.74	1.05 (0.94–1.17)	0.42
Eggs	1.02 (0.80–1.31)	0.86	1.12 (0.95–1.32)	0.17
Fish and seafood	0.99 (0.91–1.08)	0.84	0.98 (0.92–1.04)	0.50
Meat	1.02 (0.94–1.11)	0.59	1.02 (0.96–1.08)	0.53

Dietary data were self-reported. Models adjusted for all other individual food groups in addition to age, sex, total energy intake, educational attainment, smoking status, alcohol intake, margarine intake, physical activity, body mass index, total cholesterol, hypertension, diabetes, estimated glomerular filtration rate, hormone replacement therapy medication use, and statin medication use.

## Discussion

In our analysis of 3,635 African American participants in the JHS, there was no significant association between plant-based dietary patterns and CVD incidence, all-cause mortality, or CVDs analyzed separately (CHD, total stroke, ischemic stroke, and hemorrhagic stroke). This lack of an association persisted when stratifying by sex, BMI, hypertension status, and diabetes status and was observed for the overall PDI as well as hPDI and uPDI. Despite this lack of association for the dietary indices, several individual food groups were associated with CVD or mortality risk. Specifically, each additional serving of legumes was associated with a 41% reduction in CVD risk, while an additional serving of healthy oils was associated with a 10% increase in CVD risk. Additional daily servings of whole grains and SSBs were associated with a 13% and 7% increased risk for all-cause mortality, respectively.

Our results are not uniform and show a number of similarities to and differences from previous studies. In contrast to observational studies on vegetarians and vegans that have consistently found a lower risk for CVD and all-cause mortality [3,10,29,30], we did not observe this association when using PDIs to describe dietary patterns. Stratifying CVD by type, we did not observe any elevation in incident stroke risk (total, ischemic, or hemorrhagic) among participants with higher PDI scores, whereas a vegetarian diet has been previously associated with higher risk for stroke, particularly hemorrhagic stroke [31].

We modeled our 3 PDIs after those used in several other studies of American populations, including the National Health and Nutrition Examination Survey (NHANES) and the Atherosclerosis Risk in Communities (ARIC) study [7,8,32]. In ARIC participants, those with the highest versus lowest adherence to an overall plant-based dietary pattern had 8%–25% lower risk of CHD or CVD risk. Importantly, in the Nurses’ Health Study and Health Professionals Follow-Up Study, nearly all participants were white, and had lower baseline rates of hypertension and diabetes and lower BMI compared with participants in the JHS. Among NHANES participants, there was no association between CVD mortality and overall PDI, hPDI, or uPDI scores [8]. An inverse association was found only among participants with hPDI score above the median, where a 10-point higher hPDI score was associated with a 5% reduction in all-cause mortality risk. This finding suggests that health benefits related to plant-based diets may only be evident once a minimum level of plant-based eating is achieved. This observation may help to explain our results.

The quality and variability of overall diet among JHS participants are important considerations in interpreting our findings. While overall diet quality can be difficult to infer from FFQs and ranked scores like our PDIs, the Life’s Simple 7 healthy diet score is an absolute measure of dietary quality in that it uses absolute thresholds to classify participants according to their intake of specific foods and nutrients. As such, the Life’s Simple 7 healthy diet score is a useful metric for overall dietary quality that can be compared across populations. In the Life’s Simple 7 healthy diet score, a score of <2 indicates poor diet quality, 2–3 indicates intermediate diet quality, and 4–5 indicates ideal diet quality [24]. In prior investigations of the JHS cohort, 57.4% of participants were found to have poor diet quality by this metric, whereas only 0.9% met the criteria for an ideal diet [33,34]. In our study, those in the lowest overall PDI tertile met, on average, only 1.1 of the 5 Life’s Simple 7 criteria for an ideal diet. Moreover, those in the highest tertile of overall PDI met on average only 1.5 criteria, and those in the highest tertile of hPDI still met on average only 1.6 of the 5 criteria. These findings suggest poor overall diet and low variability in the diet quality of JHS participants.

The Dietary Approaches to Stop Hypertension (DASH) diet score can also be considered as a measure of overall healthfulness of participants’ diets. The DASH scores of JHS participants are also low overall. Tyson et al. investigated DASH diet adherence in the JHS and observed a median DASH score of 1.0 among participants, with 75% of participants scoring ≤1.5 on an 8-point scale [35]. By contrast, the mean DASH score observed in studies of NHANES participants was approximately 2.9 on a similar 9-point scale (which also included sodium intake scores), suggesting that NHANES participants likely have healthier diets than JHS participants [36,37]. In an urban community-based cohort, the Healthy Aging in Neighborhoods of Diversity across the Life Span (HANDLS) study, the median DASH score was 1.5 [38]. In each of these studies, black race was associated with lower DASH scores [36–38].

Our findings illuminate several important considerations when using the PDI approach to study the impact of plant-based diets on health outcomes. The use of sample-based scoring methods for scoring plant-based diets may, in part, explain the lack of association observed in our study. If there is a threshold for the effect of diet healthfulness on CVD risk, as the NHANES PDI study suggests [8], it may be that we were unable to observe such a relationship in this study by comparing intake between participants because diet quality was low on average for the entire study population. Furthermore, the PDI scores, although designed to rank participants according to degree of adherence to plant-based dietary patterns, did not capture those who exclusively consume plant-derived foods. For example, participants in the highest tertile of overall PDI still consumed, on average, 37 g of animal protein, 14 g of processed meat, 19 g of fish, and only 25 g of fiber per day. As such, future diet indices with an absolute scoring system may better represent the health impacts of a plant-based diet.

Additionally, the use of FFQs may make the diet scores more challenging to interpret because preparation methods and other dietary behaviors and preferences may not have been adequately captured in our study population. For example, it is not possible to discern whether there was high consumption of fried foods (either animal- or plant-based) in this population. In the dietary data, cooking oils were not separated from other oils in participants’ oil consumption but rather grouped under a general vegetable oils category. Prior PDIs have categorized plant-based oils as “healthy oils,” and we also used this categorization in our indices. If frying foods was a large contributor to this “healthy oils” category, it may have tempered the beneficial impacts of other plant food categories. The observation of a 10% higher CVD risk associated with each additional serving of healthy oils is consistent with this possibility that cooking and preparation methods impact the overall association of the PDIs with CVD.

The positive association between whole grain intake and mortality was also unexpected, but may be related to limited variability in the whole grain intake of the population or may suggest reverse causality. A prior analysis of dietary patterns in the JHS found that, among Life’s Simple 7 criteria for a healthy diet, whole grain intake had the lowest adherence, with only 4.1% of the JHS cohort meeting the recommendation of 3 or more 1-ounce servings per day [33]. Although we implemented measures to reduce reverse causation, we cannot exclude the possibility of reverse causation influencing our results, particularly for whole grains. A prior study investigating rates of hypertension and DASH diet scores also found an unexpected, and difficult to explain, positive association between DASH diet score and hypertension in the JHS cohort [35].

Our observed statistically significant inverse association between legumes and CVD risk, as well as the positive association between SSBs and CVD risk, is consistent with prior knowledge. Legumes are a rich source of fiber, are low in fat, and contain a variety of bioactive phytochemicals (e.g., phytate, polyphenols, and flavonoids), which can reduce blood pressure, inflammation, and risk of CVD [39]. The American Heart Association recommends consumption of plant-based sources of protein as part of an overall healthy dietary pattern for CVD prevention [40]. The added sugar and calories from added sugar in SSBs can result in weight gain [41]. Obesity is an established risk factor for the development of CVD [42].

The findings of our study should be interpreted within the context of the study strengths and limitations. One limitation of this study is the use of self-reported dietary intake, which may result in measurement error. However, the FFQ used in the JHS was developed specifically for assessing diet in American individuals residing in the southern region of the US, and calibration and validation studies in a subset of JHS participants found that it had reasonable validity and performed similarly for most nutrients when compared to both 24-hour recalls and a longer version of the Delta NIRI FFQ [14,15]. This tailoring of the FFQ and dietary index to our population’s dietary patterns is a marked strength of our study and may reduce misclassification bias [43].

While our analysis adjusted for many sociodemographic and behavioral factors and relevant medical history, this study may still be limited by residual confounding. Reverse causation, as described earlier, may also be a potential concern if participants at higher risk for CVD had intentionally adopted a more plant-based dietary pattern. Notably, prevalences of diabetes and hypertension in JHS participants at baseline were about double those in the ARIC study [9], and average BMI among JHS participants was about 3 kg/m^2^ higher [44]. However, our models adjusted for risk factors for CVD, and the consistency of results in analyses stratified by hypertension and diabetes status adds to the validity of our findings. In addition, the prospective analysis (i.e., dietary assessment preceded outcome ascertainment) and the exclusion of participants with CVD, myocardial infarction, or stroke at baseline minimizes the potential for reverse causation. Additionally, the number of incident CVD cases was relatively small (293 CVD cases out of 3,536 participants) in our study; thus, we may not have detected a statistically significant association due to low power.

This study has a number of important strengths, including a relatively large sample size comprised exclusively of African American adults, long duration of follow-up, and rigorous ascertainment of outcomes. It adds to a growing body of research to understand the association between plant-based dietary patterns and disease risk in populations reflective of the general American public. The black American population is particularly underrepresented in research, yet experiences a disproportionate burden of CVD risk factors and outcomes [45–48]. Moreover, eating patterns have cultural and regional determinants [49–51], and more research is needed to understand the role of these differing dietary patterns, to address health disparities. It is also unclear whether the components of a plant-based dietary pattern differ meaningfully among racial groups or regions, and whether specific patterns within a plant-based diet mediate the potential associations of plant-based diets with health and disease prevention. This study can begin to contextualize such questions.

### Conclusion

In summary, our results found no association of an overall, healthy, or unhealthy plant-based dietary pattern with CVD incidence or all-cause mortality in a community-based population of African American individuals in the southern region of the US who consumed a range of both plant-derived and animal-derived foods.

## Supporting information

S1 STROBE Checklist (DOCX)

S1 Fig Association between the overall plant-based diet score and incident cardiovascular disease (CVD) using the continuous score. The histogram shows the distribution of the overall plant-based diet score. The solid line represents hazard ratios for incident CVD, adjusting for age, sex, total energy intake, educational attainment, smoking status, physical activity, alcohol intake, margarine intake, diabetes, hypertension, total cholesterol, estimated glomerular filtration rate, body mass index, hormone replacement therapy medication use, and statin medication use. The dashed lines represent 95% confidence intervals.
(TIF)

S2 Fig Association between the healthy plant-based diet score and incident cardiovascular disease (CVD) using the continuous score. The histogram shows the distribution of the healthy plant-based diet score. The solid line represents hazard ratios for incident CVD, adjusting for age, sex, total energy intake, educational attainment, smoking status, physical activity, alcohol intake, margarine intake, diabetes, hypertension, total cholesterol, estimated glomerular filtration rate, body mass index, hormone replacement therapy medication use, and statin medication use. The dashed lines represent 95% confidence intervals.
(TIF)

S3 Fig Association between the unhealthy plant-based diet score and incident cardiovascular disease (CVD) using the continuous score. The histogram shows the distribution of the unhealthy plant-based diet score. The solid line represents hazard ratios for incident CVD, adjusting for age, sex, total energy intake, educational attainment, smoking status, physical activity, alcohol intake, margarine intake, diabetes, hypertension, total cholesterol, estimated glomerular filtration rate, body mass index, hormone replacement therapy medication use, and statin medication use. The dashed lines represent 95% confidence intervals.
(TIF)

S4 Fig Association between the overall plant-based diet score and all-cause mortality using the continuous score. The histogram shows the distribution of the overall plant-based diet score. The solid line represents hazard ratios for all-cause mortality, adjusting for age, sex, total energy intake, educational attainment, smoking status, physical activity, alcohol intake, margarine intake, diabetes, hypertension, total cholesterol, estimated glomerular filtration rate, body mass index, hormone replacement therapy medication use, and statin medication use. The dashed lines represent 95% confidence intervals.
(TIF)

S5 Fig Association between the healthy plant-based diet score and all-cause mortality using the continuous score. The histogram shows the distribution of the healthy plant-based diet score. The solid line represents hazard ratios for all-cause mortality, adjusting for age, sex, total energy intake, educational attainment, smoking status, physical activity, alcohol intake, margarine intake, diabetes, hypertension, total cholesterol, estimated glomerular filtration rate, body mass index, hormone replacement therapy medication use, and statin medication use. The dashed lines represent 95% confidence intervals.
(TIF)

S6 Fig Association between the unhealthy plant-based diet score and all-cause mortality using the continuous score. The histogram shows the distribution of the unhealthy plant-based diet score. The solid line represents hazard ratios for all-cause mortality, adjusting for age, sex, total energy intake, educational attainment, smoking status, physical activity, alcohol intake, margarine intake, diabetes, hypertension, total cholesterol, estimated glomerular filtration rate, body mass index, hormone replacement therapy medication use, and statin medication use. The dashed lines represent 95% confidence intervals.
(TIF)

S1 Table Food items derived from the Nutrition Data System for Research (NDSR) software and scoring of food categories in each plant-based diet index*. (DOCX)

S2 Table Selected baseline demographic, socioeconomic, and health characteristics by tertiles of healthy plant-based diet index in the Jackson Heart Study. (DOCX)

S3 Table Selected baseline demographic, socioeconomic, and health characteristics by tertiles of unhealthy plant-based diet index in the Jackson Heart Study. (DOCX)

S4 Table Baseline characteristics of the analytic sample and total study population including participants with missing covariates. (DOCX)

S5 Table Hazard ratios (95% confidence intervals) for incident cardiovascular disease (CVD) and all-cause mortality and plant-based diet indices for progressively adjusted models, comparing a complete case analysis and imputed analysis. (DOCX)

S6 Table Selected nutritional characteristics by tertiles of healthy plant-based diet index in the Jackson Heart Study. (DOCX)

S7 Table Selected nutritional characteristics by tertiles of unhealthy plant-based diet index in the Jackson Heart Study. (DOCX)

S8 Table Incidence rate and minimally adjusted hazard ratios for incident cardiovascular disease and all-cause mortality and plant-based diet indices. (DOCX)

S9 Table Hazard ratios (95% confidence intervals) for cardiovascular disease subtypes (incident coronary heart disease [CHD] and stroke) and plant-based diet indices for progressively adjusted models. (DOCX)

S10 Table Hazard ratios (95% confidence intervals) for stroke subtypes (ischemic stroke and hemorrhagic stroke) and plant-based diet indices for progressively adjusted models. (DOCX)

S11 Table Hazard ratios (95% confidence intervals) for incident cardiovascular disease (CVD) and all-cause mortality and plant-based diet indices for progressively adjusted models using quintiles (instead of tertiles). (DOCX)

S12 Table Adjusted hazard ratios* and 95% confidence intervals for incident cardiovascular disease and all-cause mortality for highest versus lowest quintile of score components of the plant-based diet index. (DOCX)

## Abbreviations

ARIC: Atherosclerosis Risk in Communities
BMI: body mass index
CHD: coronary heart disease
CVD: cardiovascular disease
DASH: Dietary Approaches to Stop Hypertension
eGFR: estimated glomerular filtration rate
FFQ: food frequency questionnaire
hPDI: healthy plant-based diet index
HR: hazard ratio
JHS: Jackson Heart Study
HRT: hormone replacement therapy
NDSR: Nutrition Data System for Research
NHANES: National Health and Nutrition Examination Survey
PDI: plant-based diet index
SSB: sugar-sweetened beverage
uPDI: unhealthy plant-based diet index
