# Supplementary figures and images for: Plant-based diets and incident cardiovascular disease and all-cause mortality in African Americans: A cohort study
Source: PLoS Med. 2022 Jan 5;19(1):e1003863. doi: 10.1371/journal.pmed.1003863 (PMC8730418; doi:10.1371/journal.pmed.1003863)

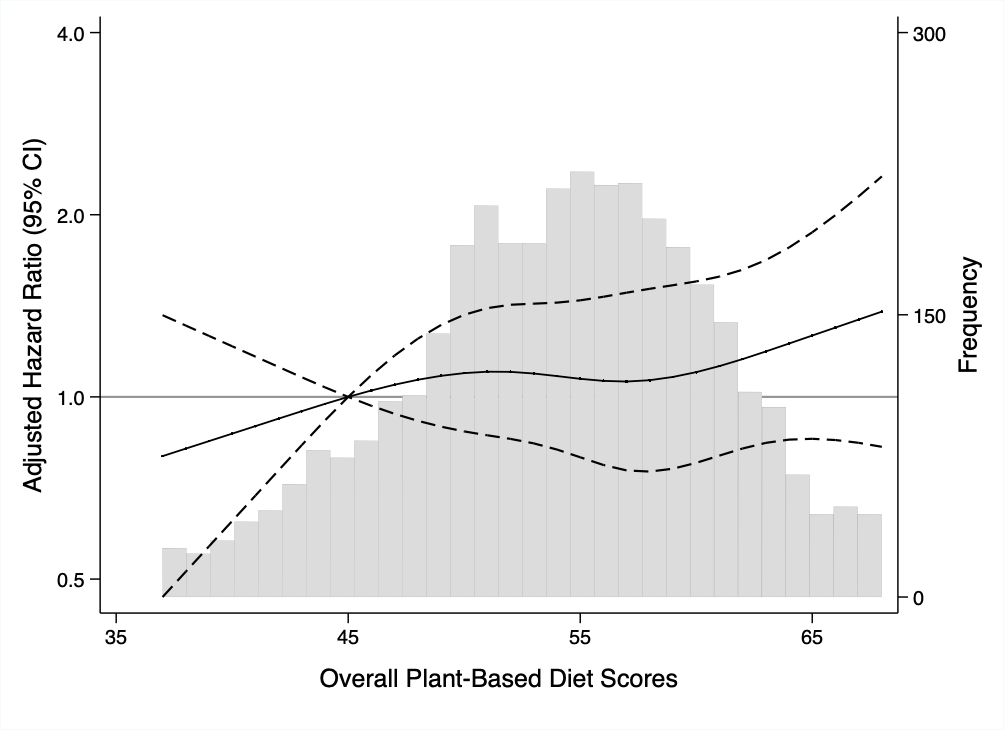

Supplement: S1 Fig — The histogram shows the distribution of the overall plant-based diet score. The solid line represents hazard ratios for incident CVD, adjusting for age, sex, total energy intake, educational attainment, smoking status, physical activity, alcohol intake, margarine intake, diabetes, hypertension, total cholesterol, estimated glomerular filtration rate, body mass index, hormone replacement therapy medication use, and statin medication use. The dashed lines represent 95% confidence intervals. (TIF) [file pmed.1003863.s002.tif]

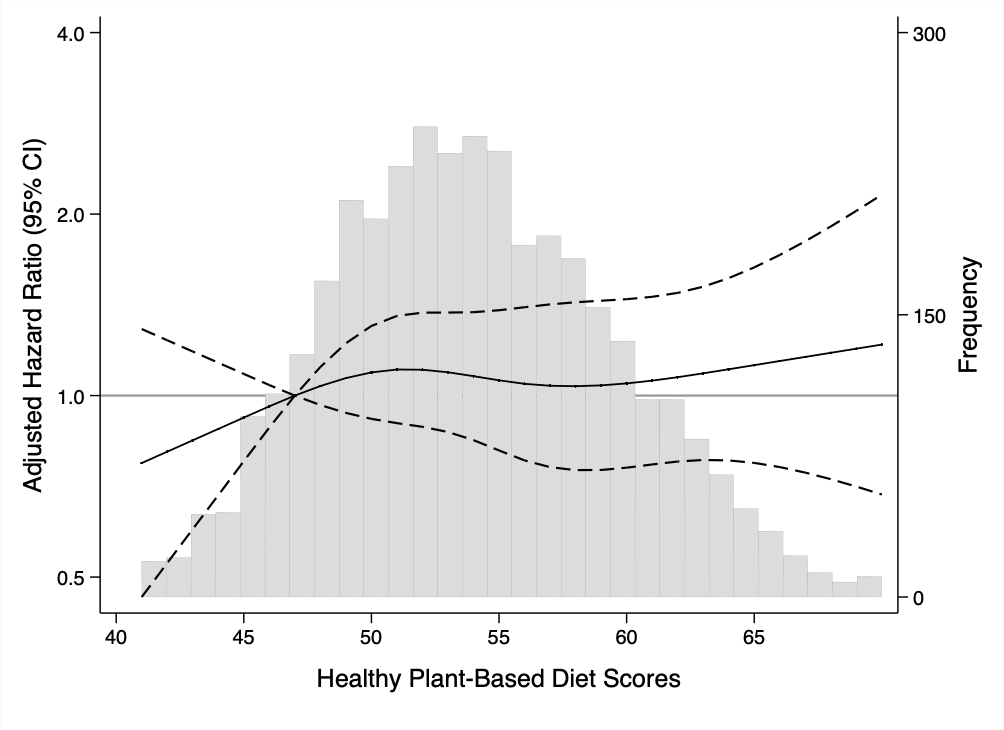

Supplement: S2 Fig — The histogram shows the distribution of the healthy plant-based diet score. The solid line represents hazard ratios for incident CVD, adjusting for age, sex, total energy intake, educational attainment, smoking status, physical activity, alcohol intake, margarine intake, diabetes, hypertension, total cholesterol, estimated glomerular filtration rate, body mass index, hormone replacement therapy medication use, and statin medication use. The dashed lines represent 95% confidence intervals. (TIF) [file pmed.1003863.s003.tif]

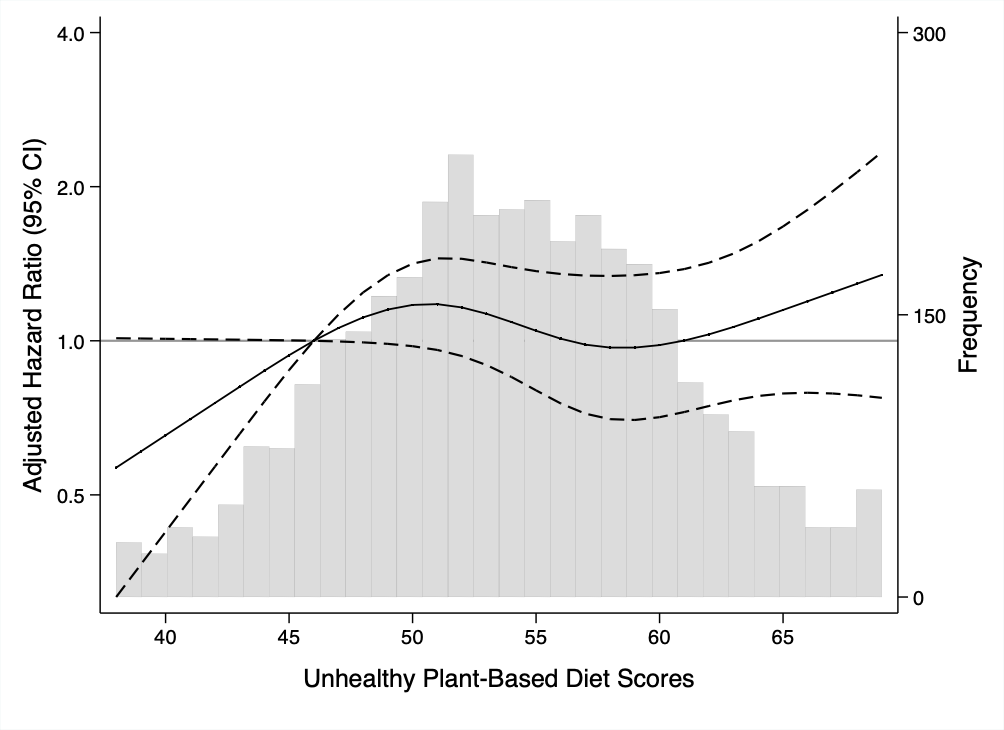

Supplement: S3 Fig — The histogram shows the distribution of the unhealthy plant-based diet score. The solid line represents hazard ratios for incident CVD, adjusting for age, sex, total energy intake, educational attainment, smoking status, physical activity, alcohol intake, margarine intake, diabetes, hypertension, total cholesterol, estimated glomerular filtration rate, body mass index, hormone replacement therapy medication use, and statin medication use. The dashed lines represent 95% confidence intervals. (TIF) [file pmed.1003863.s004.tif]

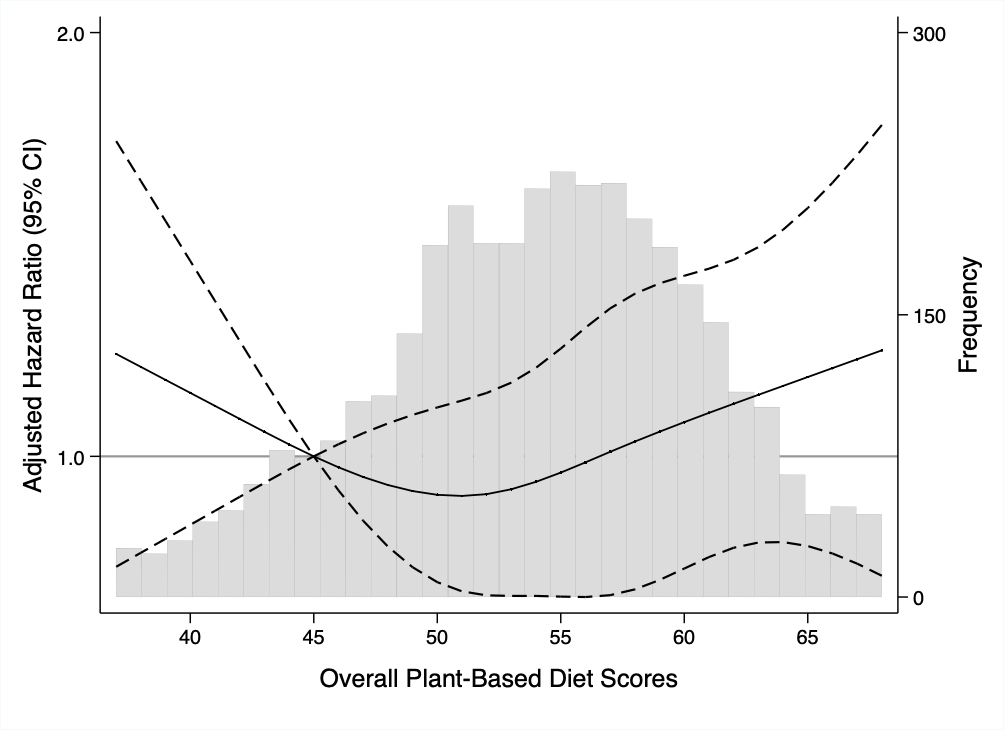

Supplement: S4 Fig — The histogram shows the distribution of the overall plant-based diet score. The solid line represents hazard ratios for all-cause mortality, adjusting for age, sex, total energy intake, educational attainment, smoking status, physical activity, alcohol intake, margarine intake, diabetes, hypertension, total cholesterol, estimated glomerular filtration rate, body mass index, hormone replacement therapy medication use, and statin medication use. The dashed lines represent 95% confidence intervals. (TIF) [file pmed.1003863.s005.tif]

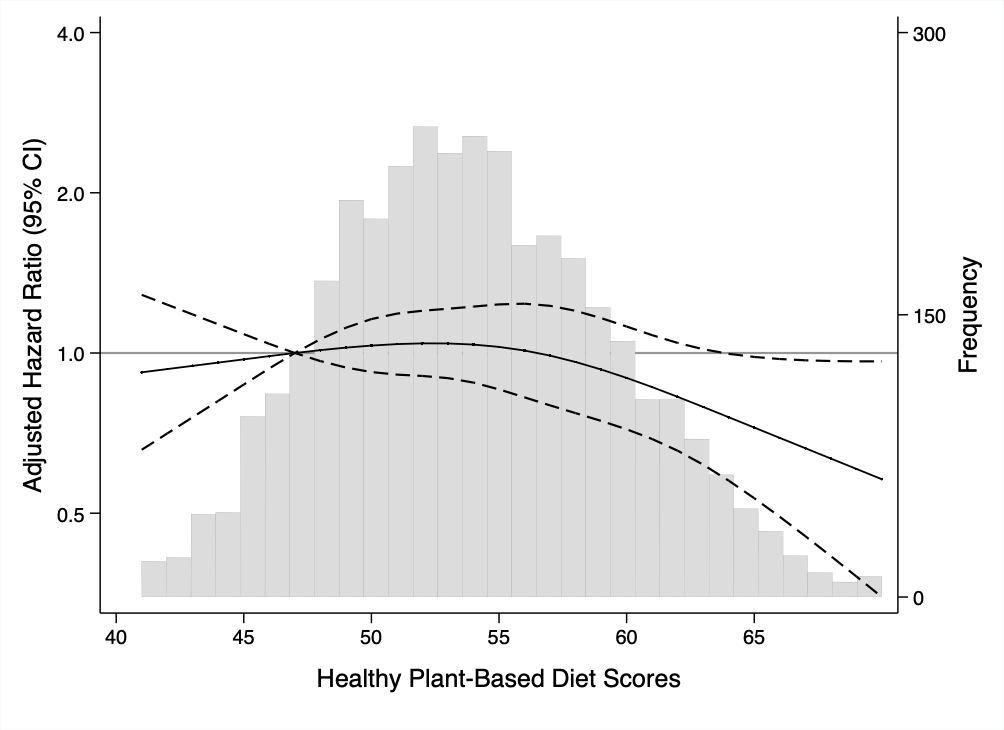

Supplement: S5 Fig — The histogram shows the distribution of the healthy plant-based diet score. The solid line represents hazard ratios for all-cause mortality, adjusting for age, sex, total energy intake, educational attainment, smoking status, physical activity, alcohol intake, margarine intake, diabetes, hypertension, total cholesterol, estimated glomerular filtration rate, body mass index, hormone replacement therapy medication use, and statin medication use. The dashed lines represent 95% confidence intervals. (TIF) [file pmed.1003863.s006.tif]

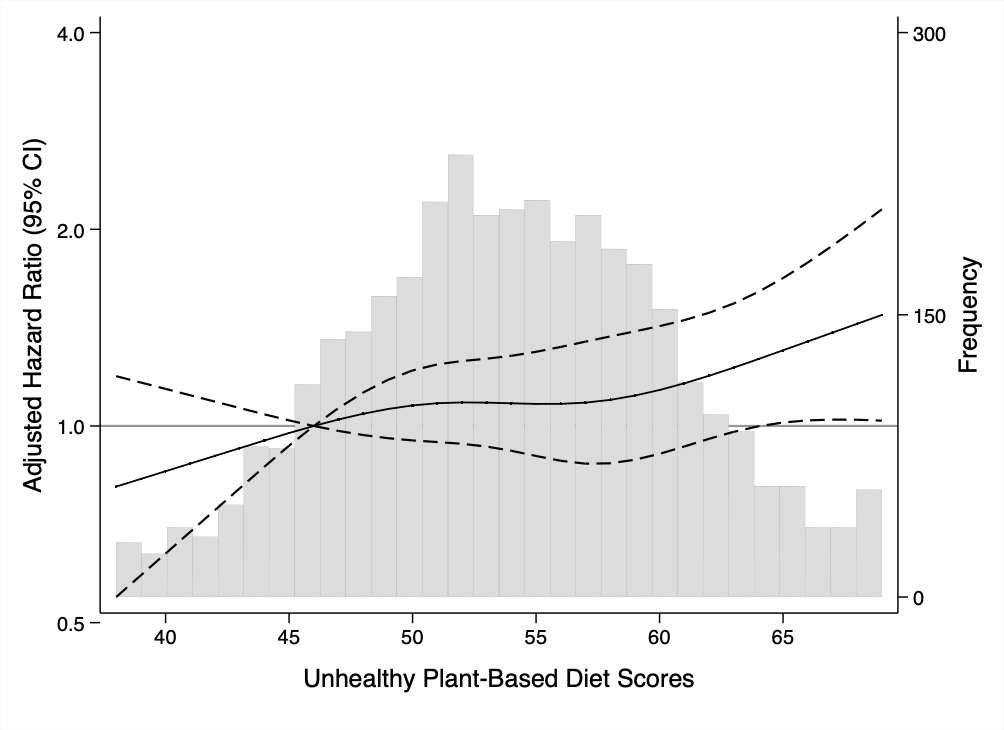

Supplement: S6 Fig — The histogram shows the distribution of the unhealthy plant-based diet score. The solid line represents hazard ratios for all-cause mortality, adjusting for age, sex, total energy intake, educational attainment, smoking status, physical activity, alcohol intake, margarine intake, diabetes, hypertension, total cholesterol, estimated glomerular filtration rate, body mass index, hormone replacement therapy medication use, and statin medication use. The dashed lines represent 95% confidence intervals. (TIF) [file pmed.1003863.s007.tif]
