# Supplementary material for: Plant-based diets and incident cardiovascular disease and all-cause mortality in African Americans: A cohort study
Source: PLoS Med. 2022 Jan 5;19(1):e1003863. doi: 10.1371/journal.pmed.1003863 (PMC8730418; doi:10.1371/journal.pmed.1003863)
Supplement: S1 Table — (DOCX) [file pmed.1003863.s008.docx]

**S1 Table. Food items derived from the Nutrition Data Systems for Research (NDSR) software and scoring of food categories in each plant-based dietary index^*^**

| *Food Groups* | | *Food Items* |  | | | *Dietary Index Scoring* | |
| --- | --- | --- | --- | --- | --- | --- | --- |
| *Healthy Plant Foods* |  | |  | ***Overall Plant-Based Diet Index*** | ***Healthy Plant-Based Diet Index*** | | ***Unhealthy Plant-Based Diet Index*** |
| Whole Grains | Grains, flour, and dry mixes (whole grain), loaf-type bread and plain rolls (whole grain), other breads (quick breads, corn muffins, tortillas—whole grain), crackers (whole grain), pasta (whole grain), ready-to-eat cereal (whole grain), popcorn, snack bars (whole grain) | |  | Positive | Positive | | Reverse |
| Fruits | Citrus fruit, fruit excluding citrus fruit, fruit-based savory snacks (apple chips, banana chips) | |  | Positive | Positive | | Reverse |
| Vegetables | Dark-green vegetables (excludes fried), deep-yellow vegetables (excludes fried), tomato, avocado and similar, other vegetables (including mixed vegetables from other categories, vegetable relishes), other starchy vegetables (cassava, corn, green peas, jicama), vegetable juice, pickled foods | |  | Positive | Positive | | Reverse |
| Nuts | Nuts and seeds, nut and seed butters | |  | Positive | Positive | | Reverse |
| Legumes | Legumes (cooked dried beans, mature lima beans), meat alternatives (tofu, tempeh, soy nuts, veggie burgers) | |  | Positive | Positive | | Reverse |
| Vegetable Oils | Oil, salad dressing (regular and reduced fat) | |  | Positive | Positive | | Reverse |
| Tea & Coffee | Tea (unsweetened), coffee (unsweetened regular and decaffeinated) | |  | Positive | Positive | | Reverse |
| *Less Healthy Plant Foods* | | |  |  |  | |  |
| Fruit Juices | Citrus juice, fruit juice excluding citrus juice | |  | Positive | Reverse | | Positive |
| Refined Grains | Grains, flour, and dry mixes (refined grain and some whole grain), loaf-type bread and plain rolls (refined grain and some whole grain), other breads (quick breads, corn muffins, tortillas—refined grain and some whole grain), crackers (refined grain and some whole grain), pasta (refined grain and some whole grain), ready-to-eat cereal (refined grain and some whole grain), snack bars (refined grain and some whole grain), snack chips (refined grain and some whole grain) | |  | Positive | Reverse | | Positive |
| Potatoes | White potatoes, fried potatoes | |  | Positive | Reverse | | Positive |
| Miscellaneous Unhealthy Plant-Based Foods | Fried fruits, fried vegetables, vegetable-based savory snack (including potato chips, onion ring chips), shortening | |  | Positive | Reverse | | Positive |
| Artificially and Sugar Sweetened Beverages | Sweetened soft drinks, sweetened fruit drinks, sweetened tea (sugar and artificial sweetener), sweetened coffee (sugar and artificial sugar, regular and decaffeinated), sweetened water | |  | Positive | Reverse | | Positive |
| Sweets & Desserts | Cakes, cookies, pies, pastries, danish, doughnuts, cobblers, frozen nondairy dessert, sugar, syrup, honey, jam, jelly, preserves, sweet sauces, chocolate candy, non-chocolate candy, frosting or glaze, miscellaneous dessert | |  | Positive | Reverse | | Positive |
| *Animal Foods* | | |  |  |  | |  |
| Animal Fats | Butter and other animal fats, gravy | |  | Reverse | Reverse | | Reverse |
| Dairy | Milk (whole, reduced fat, low fat, fat free), flavored milk beverages (sweetened or artificially sweetened), cheese (full fat, reduced fat, low fat, fat free), yogurt (whole milk, low fat, fat free, sweetened, artificially sweetened), frozen dairy dessert, pudding and other dairy dessert, cream (full fat, reduced fat, low fat, fat free) | |  | Reverse | Reverse | | Reverse |
| Eggs | Eggs | |  | Reverse | Reverse | | Reverse |
| Fish & Seafood | Fish (fresh and smoked), lean fish (fresh and smoked), fried fish, shellfish, fried shellfish | |  | Reverse | Reverse | | Reverse |
| Meat | Beef, lean beef, veal, lean veal, lamb, lean lamb, fresh pork, lean fresh pork, cured pork, lean cured pork, game, poultry, lean poultry, fried chicken, cold cuts and sausage, organ meats, meat based savory snack (including pork rinds), soup broth | |  | Reverse | Reverse | | Reverse |

**^*^** In the Jackson Heart Study, all food items in the table were derived using the NDSR software, based on participants’ responses on the food frequency questionnaire.
