# Supplementary material for: Plant-based diets and incident cardiovascular disease and all-cause mortality in African Americans: A cohort study
Source: PLoS Med. 2022 Jan 5;19(1):e1003863. doi: 10.1371/journal.pmed.1003863 (PMC8730418; doi:10.1371/journal.pmed.1003863)
Supplement: S3 Table — (DOCX) [file pmed.1003863.s010.docx]

**S3 Table.** **Selected baseline demographic, socioeconomic, and health characteristics by tertiles of unhealthy plant-based diet index in the Jackson Heart Study**

| Characteristic | Unhealthy Plant-Based Diet Index | | | |
| --- | --- | --- | --- | --- |
|  | Tertile 1  n=1,289 (35.5%) | Tertile 2  n=1,247 (34.3%) | Tertile 3  n=1,099 (30.2%) | p-value |
| Median Index Score (Range) | 48 (35-51) | 54 (52-57) | 61 (58-73) |  |
| Age, years | 54.7 (11.5) | 54.4 (12.6) | 52.1 (13.7) | <0.001 |
| Female, % | 68 | 63.7 | 59.9 | <0.001 |
| Total energy intake, kcal/day | 2286 (886) | 2136 (911) | 2368 (906) | <0.001 |
| Educational attainment |  |  |  | <0.001 |
| Less than high school/GED, % | 10.9 | 16.9 | 18.3 |  |
| High school or GED completion, % | 17.8 | 20.8 | 20.2 |  |
| Attended college or trade school, % | 71.4 | 62.3 | 61.5 |  |
| Smoking Status |  |  |  | <0.001 |
| Current smoker, % | 9.7 | 10.9 | 13.3 |  |
| Former smoker, % | 20.6 | 18.4 | 13.4 |  |
| Physical activity index ^*^ | 2.2 (0.8) | 2.1 (0.8) | 2.0 (0.8) | <0.001 |
| Alcohol intake, g/day | 3.3 (9.4) | 3.5 (10.7) | 4.9 (16.8) | 0.006 |
| Margarine intake, servings/day | 1.3 (1.8) | 1.2 (1.7) | 1.3 (1.7) | 0.538 |
| Fasting total cholesterol, mg/dl | 200 (37) | 201 (40) | 199 (39) | 0.551 |
| Hypertension, % ^†^ | 55.7 | 52.8 | 48.1 | 0.001 |
| Diabetes, % ^‡^ | 24.2 | 19.6 | 11.5 | <0.001 |
| eGFR, ml/min/1.73m^2^ ^§^ | 96.1 (20.0) | 95.3 (20.2) | 97.0 (20.8) | 0.136 |
| BMI, kg/m^2^ | 32.2 (7.0) | 31.7 (7.0) | 31.2 (7.5) | 0.002 |
| HRT medication use history, % of females | 19.0 | 17.6 | 16.9 | 0.963 |
| Statin medication use, % | 11.6 | 12.3 | 9.3 | 0.058 |
| Systolic blood pressure, mmHg | 127 (16) | 126 (17) | 127 (17) | 0.215 |
| HbA1c, mmol/mol | 42 (14.2) | 41 (12.0) | 39 (9.8) | <0.001 |
| Life’s Simple 7 Total Score ^\|\|^ | 7.4 (2.1) | 7.1 (2.1) | 7.0 (2.0) | <0.001 |

Values are means (standard deviations) for continuous variables and % for categorical variables unless otherwise noted. Statistical differences in tertiles of plant-based diet index were tested using analysis of variance for continuous variables and chi-square tests for categorical variables, with p<0.05 denoting statistical significance.

^*^ Physical activity index is a measure from 0 (low) to 5 (high) of activity in daily living.

^†^ Hypertension was defined as having blood pressure ≥ 140/90 mmHg or use of blood pressure-lowering medication within two weeks prior to the clinic visit.

^‡^ Diabetes was defined as having either fasting glucose of ≥ 126 mg/dL, HbA1c ≥ 6.5%, or use of diabetic medication within two weeks prior to the clinic visit.

^§^ eGFR was assessed using the 4-variable CKD-EPI.

^||^ Life’s Simple 7 total score is a composite score describing cardiovascular health ranging from 0 to 14 that sums American Heart Association’s description of poor (0), intermediate (1), or ideal (2) health scores for smoking, diet, physical activity level, BMI, blood pressure, total cholesterol, and fasting plasma glucose.

Abbreviations: BMI, body mass index; CKD-EPI, Chronic Kidney Disease-Epidemiology; eGFR, estimated glomerular filtration rate; GED, general educational development; HRT, hormone replacement therapy; HbA1c, hemoglobin A1c.
