# Supplementary material for: Plant-based diets and incident cardiovascular disease and all-cause mortality in African Americans: A cohort study
Source: PLoS Med. 2022 Jan 5;19(1):e1003863. doi: 10.1371/journal.pmed.1003863 (PMC8730418; doi:10.1371/journal.pmed.1003863)
Supplement: S5 Table — (DOCX) [file pmed.1003863.s012.docx]

**S5 Table. Hazard ratios (95% confidence intervals) for incident cardiovascular disease (CVD) and all-cause mortality and plant-based diet indices for progressively adjusted models, comparing a complete case analysis and imputed analysis**

|  |  | Hazard Ratios (95% confidence intervals) | | | | | | | |
| --- | --- | --- | --- | --- | --- | --- | --- | --- | --- |
|  |  | **Complete case analysis (N=3,635)** | | | | **Multiple imputation analysis (N=4,110)** | | | |
|  |  | Incident CVD | | All-Cause Mortality | | Incident CVD | | All-Cause Mortality | |
| Dietary Index |  | Per-SD higher | p-  value | Per-SD higher | p-  value | Per-SD higher | p-  value | Per-SD higher | p-  value |
| Overall Plant-Based Diet Index | median score (range) | 54  (30-76) |  | 54  (30-76) |  | 54  (31-76) |  | 54  (31-76) |  |
|  | Model 1 | 1.05  (0.93, 1.19) | 0.40 | 0.97  (0.89, 1.05) | 0.44 | 1.05  (0.93, 1.19) | 0.40 | 0.97  (0.89, 1.05) | 0.44 |
|  | Model 2 | 1.05  (0.93, 1.20) | 0.41 | 0.98  (0.89, 1.08) | 0.72 | 1.02  (0.91, 1.14) | 0.79 | 0.99  (0.91, 1.07) | 0.77 |
|  | Model 3 | 1.07  (0.94,1.22) | 0.30 | 1.03  (0.95, 1.14) | 0.45 | 1.05  (0.94,1.18) | 0.37 | 1.02  (0.94, 1.10) | 0.62 |
| Healthy Plant-Based Diet Index | median score | 54  (34-82) |  | 54  (34-82) |  | 54  (34-82) |  | 54  (34-82) |  |
|  | Model 1 | 1.06  (0.94, 1.19) | 0.36 | 0.89  (0.82, 0.97) | 0.008 | 1.06  (0.94, 1.19) | 0.36 | 0.89  (0.82, 0.97) | 0.008 |
|  | Model 2 | 1.07  (0.95, 1.20) | 0.27 | 0.93  (0.85, 1.01) | 0.095 | 1.04  (0.45, 1.16) | 0.45 | 0.94  (0.87, 1.01) | 0.13 |
|  | Model 3 | 1.04  (0.92, 1.18) | 0.52 | 0.93  (0.85, 1.02) | 0.11 | 1.03  (0.93, 1.15) | 0.58 | 0.93  (0.87, 1.01) | 0.10 |
| Unhealthy Plant-Based Diet Index | median score | 54  (31-76) |  | 54  (31-76) |  | 54  (31-76) |  | 54  (34-82) |  |
|  | Model 1 | 1.02  (0.90, 1.15) | 0.76 | 1.09  (1.00, 1.19) | 0.045 | 1.02  (0.90, 1.15) | 0.76 | 1.09  (1.00, 1.19) | 0.045 |
|  | Model 2 | 1.01  (0.89, 1.14) | 0.93 | 1.04  (0.96, 1.14) | 0.31 | 1.00  (0.89, 1.10) | 0.87 | 1.02  (0.94, 1.10) | 0.61 |
|  | Model 3 | 1.05  (0.93, 1.19) | 0.42 | 1.09 (1.001,1.19) | 0.047 | 1.05  (0.94, 1.18) | 0.36 | 1.07  (0.99,1.16) | 0.08 |

^*^Incident cardiovascular disease is a composite of coronary heart disease and/or stroke events. SD for PDI was 6.7, hPDI was 6.0, and uPDI was 6.7.

Model 1 was adjusted for age, sex, and total energy intake. We did not impute any data because there was no missing data.

Model 2 was adjusted for all the covariates in model 1 and was further adjusted for educational attainment, smoking status, alcohol intake, margarine intake, and physical activity.

Model 3 was adjusted for all the covariates in model 2 and was further adjusted for body mass index (BMI), total cholesterol, hypertension history, diabetes history, eGFR, HRT medication use history, and statin medication use.

SD, standard deviation.
