# Supplementary material for: Plant-based diets and incident cardiovascular disease and all-cause mortality in African Americans: A cohort study
Source: PLoS Med. 2022 Jan 5;19(1):e1003863. doi: 10.1371/journal.pmed.1003863 (PMC8730418; doi:10.1371/journal.pmed.1003863)
Supplement: S6 Table — (DOCX) [file pmed.1003863.s013.docx]

**S6 Table.** **Selected nutritional characteristics by tertiles of healthy plant-based diet index in the Jackson Heart Study**

| Characteristic | Healthy Plant-Based Diet Index | | | |
| --- | --- | --- | --- | --- |
|  | Tertile 1 | Tertile 2 | Tertile 3 | p-value |
| Life’s Simple 7 Healthy Diet Score ^*^ | 1.1 (0.9) | 1.3 (0.9) | 1.6 (0.9) | <0.001 |
| Total energy intake, kcal/day | 2089 (913) | 2203 (879) | 2499 (872) | <0.001 |
| Total fat, g/day | 85.0 (41.5) | 88.9 (41.3) | 101.7 (42.0) | <0.001 |
| Protein, g/day | 78.0 (38.1) | 79.2 (36.5) | 87.6 (36.7) | <0.001 |
| Alcohol, g/day | 3.2 (9.2) | 4.1 (13.6) | 4.6 (13.2) | 0.011 |
| Saturated fatty acid, g/day | 28.0 (14.4) | 27.8 (14.1) | 29.9 (13.9) | <0.001 |
| Carbohydrates, g/day | 255 (112) | 275 (113) | 315 (123) | <0.001 |
| Dietary fiber, g/day | 17.9 (8.0) | 21.8 (9.2) | 27.8 (11.5) | <0.001 |
| Fruit, servings/day | 2.2 (2.2) | 3.0 (2.8) | 3.9 (3.6) | <0.001 |
| Vegetables, servings/day | 3.2 (1.9) | 4.0 (2.4) | 4.9 (2.8) | <0.001 |
| Whole grains, servings/day | 0.7 (0.6) | 1.0 (0.8) | 1.5 (1.1) | <0.001 |
| Nuts, g/day | 3.9 (6.0) | 6.7 (9.1) | 13.1 (14.8) | <0.001 |
| Fish, g/day | 16.4 (20.5) | 20.1 (26.3) | 27.2 (38.4) | <0.001 |
| Processed meat, g/day | 22.1 (24.5) | 19.4 (24.0) | 18.7 (23.9) | 0.001 |
| Beverages, g/day | 360 (342) | 326 (344) | 285 (341) | <0.001 |
| Sweetened beverages, servings/wk | 11.9 (13.6) | 11.0 (13.7) | 10.9 (18.0) | 0.205 |
| Animal protein, g/day | 51.9 (30.1) | 50.5 (30.3) | 53.1 (31.0) | 0.118 |
| Vegetable protein, g/day | 20.1 (8.6) | 22.1 (9.0) | 26.8 (9.9) | <0.001 |
| Cholesterol, mg/day | 351 (213) | 321 (202) | 311 (182) | <0.001 |
| Monounsaturated fatty acids, g/day | 29.6 (15.8) | 30.4 (15.8) | 35.2 (16.0) | <0.001 |
| Polyunsaturated fatty acids, g/day | 17.3 (8.8) | 19.2 (9.5) | 23.4 (10.7) | <0.001 |
| Sodium, mg/day | 3353 (1546) | 3384 (1506) | 3652 (1430) | <0.001 |
| Potassium, mg/day | 2317 (1025) | 2498 (1079) | 2864 (1122) | <0.001 |
| Phosphorus, mg/day | 1123 (530) | 1175 (550) | 1328 (535) | <0.001 |
| Calcium, mg/day | 706 (347) | 744 (383) | 821 (372) | <0.001 |
| Magnesium, mg/day | 230 (90) | 256 (96) | 305 (100) | <0.001 |
| Iron, mg/day | 12.5 (5.9) | 13.1 (5.9) | 14.8 (5.8) | <0.001 |
| Vitamin A, mg/day | 6308 (3035) | 6889 (3221) | 7784 (3332) | <0.001 |
| Vitamin C, mg/day | 106 (68) | 112 (76) | 123 (92) | <0.001 |
| Folate, mg/day | 250 (110) | 273 (113) | 319 (120) | <0.001 |
| Vitamin B12, mcg/day | 5.2 (3.7) | 5.2 (3.6) | 5.4 (3.7) | 0.256 |
| Zinc, mg/day | 10.0 (5.3) | 10.3 (5.0) | 11.7 (5.1) | <0.001 |

Statistical differences were tested using analysis of variance for continuous variables with p<0.05 denoting statistical significance.

^*^Life’s Simple 7 Healthy Diet Score is a measure of adherence to five healthy dietary goals with score ranging from 0 (least healthy) to 5 (most healthy). Healthy diet score components are: fruits and vegetables, ≥4.5 cups/day; fish, ≥2 3.5-ounce servings/week; fiber-rich whole grains (≥1.1 g fiber per 10 g carbohydrate), ≥3 1-ounce servings per day; sodium, ≤1500 mg/d; and sugar-sweetened beverages, <36 fluid-ounce/week (≤450 kcal/wk). Dietary recommendations are scaled according to a 2000-kcal/d diet.
