# Supplementary material for: Plant-based diets and incident cardiovascular disease and all-cause mortality in African Americans: A cohort study
Source: PLoS Med. 2022 Jan 5;19(1):e1003863. doi: 10.1371/journal.pmed.1003863 (PMC8730418; doi:10.1371/journal.pmed.1003863)
Supplement: S7 Table — (DOCX) [file pmed.1003863.s014.docx]

**S7 Table.** **Selected nutritional characteristics by tertiles of unhealthy plant-based diet index in the Jackson Heart Study**

| Characteristic | Unhealthy Plant-Based Diet Index | | | |
| --- | --- | --- | --- | --- |
|  | Tertile 1 | Tertile 2 | Tertile 3 | p-value |
| Life’s Simple 7 Healthy Diet Score ^*^ | 1.8 (0.9) | 1.3 (0.9) | 0.8 (0.8) | <0.001 |
| Total energy intake, kcal/day | 2286 (886) | 2136 (911) | 2368 (906) | <0.001 |
| Total fat, g/day | 99.3 (42.8) | 85.7 (41.6) | 89.6 (40.8) | <0.001 |
| Protein, g/day | 90.5 (39.1) | 76.8 (37.1) | 76.3 (33.4) | <0.001 |
| Alcohol, g/day | 3.4 (8.9) | 3.7 (10.7) | 4.8 (16.2) | 0.011 |
| Saturated fatty acid, g/day | 30.8 (14.6) | 26.7 (13.8) | 28.1 (13.7) | <0.001 |
| Carbohydrates, g/day | 263 (10) | 268. (116) | 316 (126) | <0.001 |
| Dietary fiber, g/day | 25.2 (10.9) | 21.6 (10.5) | 19.9 (9.0) | <0.001 |
| Fruit, servings/day | 3.5 (3.4) | 2.9 (2.9) | 2.4 (2.5) | <0.001 |
| Vegetables, servings/day | 4.8 (2.7) | 3.8 (2.4) | 3.2 (2.0) | <0.001 |
| Whole grains, servings/day | 1.2 (0.9) | 1.0 (0.9) | 0.9 (0.9) | <0.001 |
| Nuts, g/day | 10.3 (12.7) | 7.0 (10.2) | 6.0 (10.1) | <0.001 |
| Fish, g/day | 30.7 (36.9) | 17.9 (24.8) | 13.5 (20.1) | <0.001 |
| Processed meat, g/day | 21.0 (24.9) | 19.0 (23.9) | 20.6 (23.6) | 0.102 |
| Beverages, g/day | 179 (231) | 307 (319) | 518 (388) | <0.001 |
| Sweetened beverages, servings/wk | 5.6 (7.4) | 10.2 (12.7) | 19.2 (20.5) | <0.001 |
| Animal protein, g/day | 59.8 (32.1) | 47.9 (30.1) | 46.9 (26.7) | <0.001 |
| Vegetable protein, g/day | 23.5 (9.8) | 22.0 (9.4) | 23.3 (9.4) | <0.001 |
| Cholesterol, mg/day | 383 (217) | 300 (190) | 296 (177) | <0.001 |
| Monounsaturated fatty acids, g/day | 34.3 (16.2) | 29.3 (15.6) | 31.3 (15.9) | <0.001 |
| Polyunsaturated fatty acids, g/day | 21.2 (10.2) | 18.6 (9.8) | 19.8 (9.9) | <0.001 |
| Sodium, mg/day | 3645 (1530) | 3286 (1530) | 3442 (1410) | <0.001 |
| Potassium, mg/day | 2691 (1118) | 2422 (1072) | 2538 (1087) | <0.001 |
| Phosphorus, mg/day | 1337 (574) | 1127 (532) | 1143 (494) | <0.001 |
| Calcium, mg/day | 807 (388) | 707 (350) | 750 (363) | <0.001 |
| Magnesium, mg/day | 279 (100) | 250 (102) | 258 (96) | <0.001 |
| Iron, mg/day | 13.8 (6.0) | 12.9 (6.0) | 13.7 (5.9) | <0.001 |
| Vitamin A, mg/day | 7603 (3146) | 6839 (3182) | 6388.(3323) | <0.001 |
| Vitamin C, mg/day | 98.9 (58.1) | 109.8 (76.8) | 134.2 (97.2) | <0.001 |
| Folate, mg/day | 297 (120) | 270 (118) | 271 (113) | <0.001 |
| Vitamin B12, mcg/day | 6.0 (4.1) | 4.8 (3.2) | 4.8 (3.5) | <0.001 |
| Zinc, mg/day | 11.6 (5.3) | 10.1 (5.2) | 10.1 (4.8) | <0.001 |

Statistical differences were tested using analysis of variance for continuous variables with p<0.05 denoting statistical significance.

^*^ Life’s Simple 7 Healthy Diet Score is a measure of adherence to five healthy dietary goals with score ranging from 0 (least healthy) to 5 (most healthy). Healthy diet score components are: fruits and vegetables, ≥4.5 cups/day; fish, ≥2 3.5-ounce servings/week; fiber-rich whole grains (≥1.1 g fiber per 10 g carbohydrate), ≥3 1-ounce servings per day; sodium, ≤1500 mg/d; and sugar-sweetened beverages, <36 fluid-ounce/week (≤450 kcal/wk). Dietary recommendations are scaled according to a 2000-kcal/d diet.
