# Supplementary material for: Plant-based diets and incident cardiovascular disease and all-cause mortality in African Americans: A cohort study
Source: PLoS Med. 2022 Jan 5;19(1):e1003863. doi: 10.1371/journal.pmed.1003863 (PMC8730418; doi:10.1371/journal.pmed.1003863)
Supplement: S8 Table — (DOCX) [file pmed.1003863.s015.docx]

**S8 Table.** **Incidence rate and minimally adjusted hazard ratios for incident cardiovascular disease and all-cause mortality and plant-based diet indices**

|  |  | | Incident Cardiovascular Disease^*^ | | All-Cause Mortality | |
| --- | --- | --- | --- | --- | --- | --- |
|  |  |  | IR per 1,000 PY (95% CI) | HR  (95% CI) | IR per 10,000 PY (95% CI) | HR  (95% CI) |
|  |  | Overall | 6.9 (6.1-7.6) |  | 11.2 (10.4-12.2) |  |
| Overall Plant-Based Diet Index | Minimally adjusted | Tertile 1 | 6.2 (5.0-7.6) | 1 (ref) | 10.7 (9.4-12.4) | 1 (ref) |
|  |  | Tertile 2 | 6.9 (5.7-8.4) | 1.02 (0.76-1.36) | 11.0 (9.6-12.7) | 0.87 (0.71-1.06) |
|  |  | Tertile 3 | 7.4 (6.1-9.0) | 1.06 (0.79-1.43) | 12.0 (10.4-13.8) | 0.93 (0.76-1.14) |
|  |  | p-trend |  | 0.69 |  | 0.49 |
| Healthy Plant-Based Diet Index | Minimally adjusted | Tertile 1 | 6.7 (5.5-8.7) | 1 (ref) | 10.6 (9.3-12.2) | 1 (ref) |
|  |  | Tertile 2 | 6.7 (5.5-8.3) | 0.97 (0.73-1.29) | 13.6 (11.9-15.5) | 1.24 (1.02-1.50) |
|  |  | Tertile 3 | 7.2 (5.9-8.7) | 1.06 (0.80-1.41) | 9.7 (8.3-11.3) | 0.87 (0.70-1.07) |
|  |  | p-trend |  | 0.68 |  | 0.21 |
| Unhealthy Plant-Based Diet Index | Minimally adjusted | Tertile 1 | 6.8 (5.6-8.2) | 1 (ref) | 10.3 (8.9-11.9) | 1 (ref) |
|  |  | Tertile 2 | 7.5 (6.2-9.0) | 1.07 (0.82-1.41) | 12.2 (10.7-13.9) | 1.17 (0.96-1.42) |
|  |  | Tertile 3 | 6.2 (4.9-7.7) | 0.97 (0.72-1.30) | 11.2 (9.7-13.1) | 1.14 (0.93-1.40) |
|  |  | p-trend |  | 0.85 |  | 0.20 |

^*^Incident cardiovascular disease is a composite of coronary heart disease and/or stroke events

Hazard ratios adjusted for age, sex, and total energy intake. P-for trend was calculated using the median value of each tertile. Incidence rate is expressed per 100,000 person-years (PY). P-trend for hazard ratios calculated using a continuous model based on median tertile values.
