# Supplementary material for: Plant-based diets and incident cardiovascular disease and all-cause mortality in African Americans: A cohort study
Source: PLoS Med. 2022 Jan 5;19(1):e1003863. doi: 10.1371/journal.pmed.1003863 (PMC8730418; doi:10.1371/journal.pmed.1003863)
Supplement: S9 Table — (DOCX) [file pmed.1003863.s016.docx]

**S9 Table. Hazard ratios (95% confidence intervals) for cardiovascular disease subtypes (incident coronary heart disease (CHD) and stroke) and plant-based diet indices for progressively adjusted models**

|  |  | Hazard Ratios (95% confidence intervals) | | | | | | | | | | | |
| --- | --- | --- | --- | --- | --- | --- | --- | --- | --- | --- | --- | --- | --- |
|  |  | Incident Coronary Heart Disease | | | | | | Stroke | | | | | |
| Dietary Index |  | Tertile 1 (ref) | Tertile 2 | Tertile 3 | p-trend | Per-SD higher^*^ | p-  value | Tertile 1 (ref) | Tertile 2 | Tertile 3 | p-trend | Per-SD higher^*^ | p-  value |
| Overall Plant-Based Diet Index | median score | 48 | 55 | 61 |  |  |  | 48 | 55 | 61 |  |  |  |
|  | cases/N | 52/1237 | 61/1258 | 60/1140 |  |  |  | 46/1237 | 52/1258 | 50/1140 |  |  |  |
|  | person-years | 14682 | 14989 | 13663 |  |  |  | 14748 | 15027 | 13717 |  |  |  |
|  | Model 1 | 1 | 1.03  (0.71-1.51) | 1.09  (0.74-1.61) | 0.65 | 1.08  (0.92, 1.27) | 0.94 | 1 | 0.99  (0.66-1.49) | 1.02  (0.67-1.54) | 0.94 | 1.01  (0.85, 1.20) | 0.91 |
|  | Model 2 | 1 | 0.97  (0.67-1.43) | 1.09  (0.74-1.61) | 0.66 | 1.08  (0.91, 1.27) | 0.37 | 1 | 0.97  (0.64-1.45) | 1.00  (0.66-1.52) | 0.99 | 1.01  (0.85, 1.21) | 0.91 |
|  | Model 3 | 1 | 1.02  (0.69-1.51) | 1.13  (0.76-1.69) | 0.54 | 1.10  (0.93,1.31) | 0.25 | 1 | 0.98  (0.65-1.49) | 0.98  (0.64-1.50) | 0.93 | 1.00  (0.84, 1.20) | 0.97 |
| Healthy Plant-Based Diet Index | median score | 48 | 54 | 60 |  |  |  | 48 | 54 | 60 |  |  |  |
|  | cases/N | 61/1295 | 51/1155 | 61/1185 |  |  |  | 55/1295 | 44/1155 | 49/1185 |  |  |  |
|  | person-years | 15393 | 13660 | 14282 |  |  |  | 15423 | 13767 | 14302 |  |  |  |
|  | Model 1 | 1 | 0.92  (0.64-1.34) | 1.10  (0.76-1.59) | 0.60 | 1.09  (0.94, 1.28) | 0.24 | 1 | 0.86  (0.58-1.28) | 0.94  (0.63-1.39) | 0.73 | 0.97  (0.82, 1.15) | 0.76 |
|  | Model 2 | 1 | 0.93  (0.63-1.35) | 1.11  (0.76-1.61) | 0.60 | 1.12  (0.95, 1.30) | 0.17 | 1 | 0.86  (0.58-1.30) | 0.91  (0.61-1.36) | 0.64 | 0.97  (0.82, 1.15) | 0.72 |
|  | Model 3 | 1 | 0.92  (0.63-1.35) | 1.04  (0.72-1.53) | 0.82 | 1.07  (0.92, 1.27) | 0.34 | 1 | 0.83  (0.55-1.26) | 0.86  (0.46-1.29) | 0.45 | 0.94  (0.79, 1.12) | 0.49 |
| Unhealthy Plant-Based Diet Index | median score | 48 | 54 | 61 |  |  |  | 48 | 54 | 61 |  |  |  |
|  | cases/N | 61/1289 | 73/1247 | 39/1099 |  |  |  | 48/1289 | 50/1247 | 50/1099 |  |  |  |
|  | person-years | 15402 | 14708 | 13224 |  |  |  | 15423 | 14842 | 13227 |  |  |  |
|  | Model 1 | 1 | 1.21  (0.86-1.70) | 0.79  (0.53-1.18) | 0.29 | 0.95  (0.82, 1.12) | 0.59 | 1 | 1.05  (0.71-1.56) | 1.28  (0.87-1.92) | 0.21 | 1.15  (0.97, 1.36) | 0.12 |
|  | Model 2 | 1 | 1.21  (0.86-1.72) | 0.79  (0.52-1.18) | 0.28 | 0.95  (0.81, 1.11) | 0.50 | 1 | 1.05  (0.70-1.57) | 1.26  (0.84-1.89) | 0.25 | 1.13  (0.95, 1.35) | 0.15 |
|  | Model 3 | 1 | 1.26  (0.88-1.79) | 0.86  (0.56-1.31) | 0.53 | 1.00  (0.85, 1.18) | 0.97 | 1 | 1.07  (0.71-1.62) | 1.36  (0.90-2.06) | 0.14 | 1.18  (0.99, 1.41) | 0.07 |

* SD for PDI was 6.7, hPDI was 6.0, and uPDI was 6.7.

Model 1 was adjusted for age, sex, and total energy intake.

Model 2 was adjusted for all the covariates in model 1 and was further adjusted for educational attainment, smoking status, alcohol intake, margarine intake, and physical activity.

Model 3 was adjusted for all the covariates in model 2 and was further adjusted for body mass index (BMI), total cholesterol, hypertension history, diabetes history, eGFR, HRT medication use history, and statin medication use.

SD, standard deviation
