# Supplementary material for: Plant-based diets and incident cardiovascular disease and all-cause mortality in African Americans: A cohort study
Source: PLoS Med. 2022 Jan 5;19(1):e1003863. doi: 10.1371/journal.pmed.1003863 (PMC8730418; doi:10.1371/journal.pmed.1003863)
Supplement: S10 Table — (DOCX) [file pmed.1003863.s017.docx]

**S10 Table. Hazard ratios (95% confidence intervals) for stroke subtypes (ischemic stroke and hemorrhagic stroke) and plant-based diet indices for progressively adjusted models**

|  |  | Hazard Ratios (95% confidence intervals) | | | | | | | |
| --- | --- | --- | --- | --- | --- | --- | --- | --- | --- |
|  |  | Ischemic stroke | | | | | | Hemorrhagic stroke | |
| Dietary Index |  | Tertile 1 (ref) | Tertile 2 | Tertile 3 | p-trend | Per-SD higher^*^ | p-  value | Per-SD higher^*^ | p-  value |
| Overall Plant-Based Diet Index | median score | 48 | 55 | 61 |  |  |  |  |  |
|  | cases/N | 43/1237 | 46/1258 | 46/1140 |  |  |  | 12/3635 |  |
|  | person-years | 14748 | 15027 | 13717 |  |  |  | 43493 |  |
|  | Model 1 | 1 | 0.93  (0.68-1.43) | 1.00  (0.65-1.54) | 0.99 | 0.99  (0.83, 1.19) | 0.94 | 1.23  (0.67, 2.25) | 0.50 |
|  | Model 2 | 1 | 0.90  (0.59-1.39) | 0.98  (0.64-1.52) | 0.94 | 0.99  (0.82, 1.20) | 0.94 | 1.18  (0.65, 2.15) | 0.59 |
|  | Model 3 | 1 | 0.94  (0.61-1.46) | 0.97  (0.62-1.51) | 0.88 | 0.99  (0.82,1.20) | 0.90 | 1.18  (0.63, 2.20) | 0.60 |
| Healthy Plant-Based Diet Index | median score | 48 | 54 | 60 |  |  |  |  |  |
|  | cases/N | 50/1295 | 43/1155 | 42/1185 |  |  |  | 12/3635 |  |
|  | person-years | 15423 | 13767 | 14302 |  |  |  | 43493 |  |
|  | Model 1 | 1 | 0.92  (0.61-1.39) | 0.88  (0.58-1.35) | 0.57 | 0.95  (0.79, 1.13) | 0.57 | 1.41  (0.81, 2.45) | 0.22 |
|  | Model 2 | 1 | 0.93  (0.62-1.41) | 0.86  (0.56-1.32) | 0.48 | 0.95  (0.79, 1.13) | 0.54 | 1.37  (0.78, 2.43) | 0.27 |
|  | Model 3 | 1 | 0.88  (0.58-1.35) | 0.79  (0.51-1.22) | 0.29 | 0.91  (0.76, 1.09) | 0.32 | 1.46  (0.81, 2.64) | 0.21 |
| Unhealthy Plant-Based Diet Index | median score | 48 | 54 | 61 |  |  |  |  |  |
|  | cases/N | 45/1289 | 46/1247 | 44/1099 |  |  |  | 12/3635 |  |
|  | person-years | 15423 | 14842 | 13227 |  |  |  | 43493 |  |
|  | Model 1 | 1 | 1.02  (0.68-1.55) | 1.20  (0.79-1.83) | 0.38 | 1.12  (0.94, 1.34) | 0.21 | 1.37  (0.77, 2.47) | 0.29 |
|  | Model 2 | 1 | 1.02  (0.67-1.56) | 1.17  (0.77-1.79) | 0.46 | 1.11  (0.92, 1.32) | 0.28 | 1.43  (0.79, 2.59) | 0.24 |
|  | Model 3 | 1 | 1.05  (0.69-1.62) | 1.26  (0.81-1.94) | 0.30 | 1.15  (0.95, 1.38) | 0.15 | 1.52  (0.82, 2.82) | 0.18 |

* SD for PDI was 6.7, hPDI was 6.0, and uPDI was 6.7. Of 148 total stroke cases, 135 cases were ischemic stroke cases, 12 were hemorrhagic stroke cases, and 1 did not have information on the stroke subtype.

Model 1 was adjusted for age, sex, and total energy intake.

Model 2 was adjusted for all the covariates in model 1 and was further adjusted for educational attainment, smoking status, alcohol intake, margarine intake, and physical activity.

Model 3 was adjusted for all the covariates in model 2 and was further adjusted for body mass index (BMI), total cholesterol, hypertension history, diabetes history, eGFR, HRT medication use history, and statin medication use.
