# Supplementary material for: Plant-based diets and incident cardiovascular disease and all-cause mortality in African Americans: A cohort study
Source: PLoS Med. 2022 Jan 5;19(1):e1003863. doi: 10.1371/journal.pmed.1003863 (PMC8730418; doi:10.1371/journal.pmed.1003863)
Supplement: S11 Table — (DOCX) [file pmed.1003863.s018.docx]

**S11 Table.** Hazard ratios (95% confidence intervals) for incident cardiovascular disease (CVD) and all-cause mortality and plant-based diet indices for progressively adjusted models using quintiles (instead of tertiles)

|  |  | Hazard Ratios (95% confidence intervals) | | | | | | | | | | | |
| --- | --- | --- | --- | --- | --- | --- | --- | --- | --- | --- | --- | --- | --- |
|  |  | Incident Cardiovascular Disease | | | | | | All-Cause Mortality | | | | | |
| Dietary Index |  | Quintile 1 (ref) | Quintile 2 | Quintile 3 | Quintile 4 | Quintile 5 | p-trend | Quintile 1 (ref) | Quintile 2 | Quintile 3 | Quintile 4 | Quintile 5 | p-  value |
| Overall Plant-Based Diet Index | median score | 46 | 51 | 55 | 58 | 63 |  | 46 | 51 | 55 | 58 | 63 |  |
|  | cases/N | 63/842 | 55/771 | 50/662 | 66/773 | 59/587 |  | 126/842 | 122/771 | 109/662 | 132/773 | 108/587 |  |
|  | person-years | 9884 | 9051 | 7814 | 9101 | 6914 |  | 12283 | 11279 | 9674 | 11257 | 8577 |  |
|  | Model 1 | 1 | 0.81  (0.56- 1.17) | 0.82  (0.56-1.20) | 0.99  (0.69-1.42) | 1.06  (0.73-1.54) | 0.50 | 1 | 0.83  (0.65-1.08) | 0.83  (0.63-1.08) | 0.95  (0.73-1.22) | 0.89  (0.68-1.16) | 0.68 |
|  | Model 2 | 1 | 0.78  (0.54-1.13) | 0.77  (0.52-1.14) | 0.95  (0.66-1.37) | 1.06  (0.73-1.54) | 0.51 | 1 | 0.82  (0.64-1.06) | 0.80  (0.61-1.04) | 0.93  (0.72-1.21) | 0.93  (0.71-1.21) | 0.89 |
|  | Model 3 | 1 | 0.90  (0.62-1.31) | 0.83  (0.56-1.24) | 1.00  (0.69-1.45) | 1.18  (0.80-1.73) | 0.35 | 1 | 0.88  (0.68-1.15) | 0.87  (0.66-1.15) | 1.04  (0.80-1.36) | 1.08  (0.82-1.43) | 0.34 |
| Healthy Plant-Based Diet Index | median score | 47 | 51 | 54 | 57 | 62 |  | 47 | 51 | 54 | 57 | 62 |  |
|  | cases/N | 67/865 | 54/680 | 56/718 | 54/713 | 62/659 |  | 133/865 | 133/680 | 140/718 | 101/713 | 90/659 |  |
|  | person-years | 10135 | 7907 | 8475 | 8390 | 7858 |  | 12598 | 9816 | 10357 | 10487 | 9813 |  |
|  | Model 1 | 1 | 0.93  (0.65-1.33) | 0.96  (0.67-1.38) | 0.96  (0.67-1.38) | 1.15  (0.81-1.65) | 0.43 | 1 | 1.12  (0.88-1.43) | 1.21  (0.95-1.53) | 0.87  (0.67-1.14) | 0.78  (0.59-1.03) | 0.03 |
|  | Model 2 | 1 | 0.97  (0.67-1.39) | 1.01  (0.70-1.45) | 1.00  (0.69-1.45) | 1.21  (0.84-1.75) | 0.31 | 1 | 1.22  (0.96-1.56) | 1.27  (0.99-1.61) | 0.96  (0.74-1.25) | 0.89  (0.67-1.17) | 0.21 |
|  | Model 3 | 1 | 0.97  (0.67-1.41) | 1.00  (0.69-1.45) | 0.94  (0.65-1.37) | 1.14  (0.78-1.65) | 0.58 | 1 | 1.27  (0.99-1.63) | 1.32  (1.02-1.68) | 0.94  (0.73-1.23) | 0.93  (0.70-1.24) | 0.27 |
| Unhealthy Plant-Based Diet Index | median score | 46 | 51 | 54 | 58 | 63 |  | 46 | 51 | 54 | 58 | 63 |  |
|  | cases/N | 60/749 | 62/775 | 78/809 | 48/718 | 45/584 |  | 116/749 | 122/775 | 141/809 | 120/718 | 98/584 |  |
|  | person-years | 8807 | 9103 | 9429 | 8509 | 6917 |  | 10973 | 11300 | 11776 | 10495 | 8525 |  |
|  | Model 1 | 1 | 1.00  (0.70-1.42) | 1.21  (0.86-1.70) | 0.82  (0.59-1.19) | 1.11  (0.76-1.65) | 0.99 | 1 | 1.04  (0.81-1.35) | 1.14  (0.89-1.46) | 1.06  (0.82-1.37) | 1.28  (0.98-1.69) | 0.09 |
|  | Model 2 | 1 | 0.96  (0.67-1.38) | 1.19  (0.84-1.67) | 0.79  (0.54-1.15) | 1.07  (0.72-1.58) | 0.83 | 1 | 0.99  (0.77-1.29) | 1.07  (0.83-1.37) | 0.97  (0.75-1.25) | 1.14  (0.86-1.50) | 0.49 |
|  | Model 3 | 1 | 1.07  (0.74-1.55) | 1.29  (0.91-1.83) | 0.87  (0.59-1.30_ | 1.25  (0.84-1.88) | 0.61 | 1 | 1.11  (0.85-1.44) | 1.21  (0.94-1.56) | 1.10  (0.84-1.43) | 1.31  (0.99-1.73) | 0.10 |

^*^Incident cardiovascular disease is a composite of coronary heart disease and/or stroke events. SD for PDI was 6.7, hPDI was 6.0, and uPDI was 6.7.

Model 1 was adjusted for age, sex, and total energy intake.

Model 2 was adjusted for all the covariates in model 1 and was further adjusted for educational attainment, smoking status, alcohol intake, margarine intake, and physical activity.

Model 3 was adjusted for all the covariates in model 2 and was further adjusted for body mass index (BMI), total cholesterol, hypertension history, diabetes history, eGFR, HRT medication use history, and statin medication use.
