# Supplementary material for: Plant-based diets and incident cardiovascular disease and all-cause mortality in African Americans: A cohort study
Source: PLoS Med. 2022 Jan 5;19(1):e1003863. doi: 10.1371/journal.pmed.1003863 (PMC8730418; doi:10.1371/journal.pmed.1003863)
Supplement: S12 Table — (DOCX) [file pmed.1003863.s019.docx]

**S12 Table.** **Adjusted hazard ratios^*^ and 95% confidence intervals for incident cardiovascular disease and all-cause mortality for highest vs. lowest quintiles of score components of the plant-based diet index**

| Score Components of Plant-Based Diet Index | Incident Cardiovascular Disease | | All-Cause Mortality | |
| --- | --- | --- | --- | --- |
|  | Hazard Ratios  (95% CI) | p-trend | Hazard Ratios  (95% CI) | p-trend |
| **Minimally adjusted** |  |  |  |  |
| Healthy Plant Foods | 0.96 (0.64-1.42) | 0.83 | 0.69 (0.52-0.90) | 0.04 |
| Unhealthy Plant Foods | 0.67 (0.43-1.03) | 0.20 | 1.07 (0.79-1.44) | 0.96 |
| Animal-Based Foods | 0.88 (0.58-1.33) | 0.63 | 1.01 (0.76-1.33) | 0.89 |
| **Fully adjusted** |  |  |  |  |
| Healthy Plant Foods | 0.98 (0.65-1.49) | 0.93 | 0.83 (0.62-1.10) | 0.64 |
| Unhealthy Plant Foods | 0.75 (0.47-1.19) | 0.49 | 1.18 (0.85-1.63) | 0.42 |
| Animal-Based Foods | 0.84 (0.54-1.31) | 0.58 | 0.98 (0.73-1.32) | 0.97 |

^*^models adjusted for other score component categories in addition to age, sex, total energy intake, educational attainment, smoking status, alcohol intake, margarine intake, physical activity, BMI, total cholesterol, hypertension history, diabetes history, eGFR, HRT medication use history, and statin medication use. P for trend was calculated using quintiles of energy-adjusted food group score components.

Abbreviations: BMI, body mass index; eGFR, estimated glomerular filtration rate; HRT, hormone replacement therapy.
